# Autophagy induces hair follicle stem cell activation and hair follicle regeneration by regulating glycolysis

**DOI:** 10.1186/s13578-023-01177-2

**Published:** 2024-01-05

**Authors:** Pingping Sun, Zhan Wang, Sixiao Li, Jiajing Yin, Yuyang Gan, Shizhao Liu, Zhen Lin, Hailin Wang, Zhexiang Fan, Qian Qu, Zhiqi Hu, Kaitao Li, Yong Miao

**Affiliations:** Department of Plastic and Aesthetic Surgery, Nanfang Hospital of Southern Medical University, Guangzhou, China

**Keywords:** Autophagy, Glycolysis, Hair follicle regeneration, Hair follicle stem cells

## Abstract

**Background:**

Hair follicle stem cells (HFSCs) typically remain quiescent and are activated only during the transition from telogen to anagen to ensure that the hair follicle enters a new cycle. The metabolic behavior of stem cells in tissues is regulated by macroautophagy/autophagy, and changes in HFSC metabolism directly affect their activation and maintenance. However, the role of autophagy in the regulation of HFSC metabolism and function remains unclear.

**Methods:**

Back skin samples were obtained from mice at different hair follicle cycle stages, and immunofluorescence staining was used to monitor autophagy in HFSCs. Mouse and human hair follicles were treated with rapamycin (Rapa, an autophagy activator) or 3-methyladenine (3-MA, an autophagy inhibitor). The effects of autophagy on the hair follicle cycle and HFSC were investigated by imaging, cell proliferation staining, and HFSC-specific marker staining. The influence and mechanism of autophagy on HFSC metabolism were explored using RNA sequencing, real-time polymerase chain reaction, immunohistochemical staining, and detection of lactate and glucose concentrations. Finally, the influence of autophagy-induced glycolysis on HFSC and the hair follicle cycle was verified by stem cell characteristics and in vivo functional experiments.

**Results:**

Autophagy in HFSC was highest during the transition from telogen to anagen. Inhibiting autophagy with 3-MA led to early entry into catagen and prolonged telogen, whereas Rapa promoted autophagy and hair growth. Autophagy activated HFSC by increasing the expression and activity of HFSC lactate dehydrogenase (Ldha), thereby transforming HFSC metabolism into glycolysis. Inhibition of Ldha expression counteracted the effects of autophagy.

**Conclusions:**

Autophagy activated HFSC by promoting the transition from HFSC metabolism to glycolysis, ultimately initiating the hair follicle cycle and promoting hair growth.

**Graphical Abstract:**

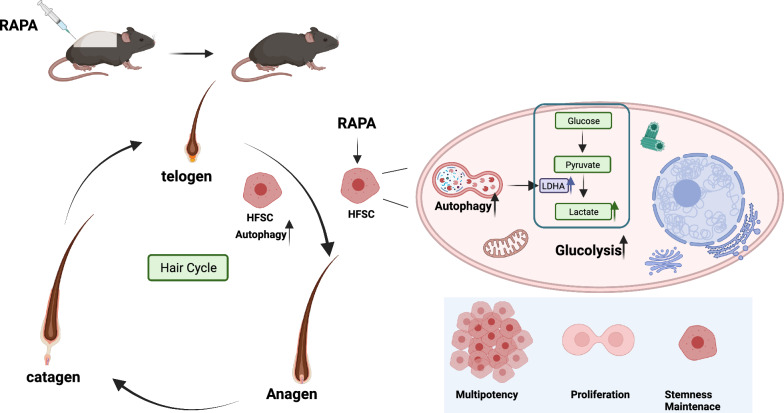

**Supplementary Information:**

The online version contains supplementary material available at 10.1186/s13578-023-01177-2.

## Introduction

Hair follicles undergo anagen, catagen, and telogen, forming a cycle of hair growth, shedding, and regrowth. Hair follicle stem cells (HFSCs) are located in the bulge zone of hair follicles and several molecular markers are used to identify bulge HFSCs, such as cytokeratin-15 (CK15) and cytokeratin-19 (CK19) promoters’ activity [1]. HFSCs undergo activation and quiescence, which are highly synchronized with the hair follicle cycle [1, 2]. Prolonged telogen and hair miniaturization caused by HFSC activation disorders are pathological conditions in several hair loss diseases, such as androgenetic alopecia (AGA) and stress-induced alopecia [3, 4]. HFSC activation is regulated by internal and external mechanisms. According to recent studies, compared to other epidermal cells, HFSCs tend to utilize glycolytic metabolism to produce more lactate, which allows them to respond quickly to hair follicle growth stimulation signals [5]. The transition from HFSC to outer root sheath (ORS) progenitor cells involves the activation of oxidative phosphorylation and the entry of glutamine into the tricarboxylic acid (TCA) cycle. Importantly, inhibiting the metabolic transition from glycolysis to oxidative phosphorylation (OXPHOS) and glutamine metabolism during the early development of the HFSC lineage can reprogram ORS progenitor cells and restore their stem cell properties, thus forming a new HFSC ecological niche [6]. However, little is known about the regulation of metabolic pathways in HFSCs.

Autophagy is an intracellular degradation system that transmits cytoplasmic substances to lysosomes for degradation and plays various roles in cellular pathological processes. Orhon et al. used a three-dimensional organoid system to evaluate the effect of autophagy on mouse salivary gland stem cells (SGSCs), in which enhanced autophagy induced SGSCs activation and played an important role in maintaining their self-renewal characteristics [7]. Several studies have shown that autophagy regulates cellular metabolism. For example, autophagy maintains mitochondrial function and quantity in hematopoietic stem cells and cellular metabolism at low levels of OXPHOS, while impaired autophagy leads to excessive activation of hematopoietic stem cells and senescence phenotypes [8].

Recent studies have shown that ɑ-ketoglutarate (ɑ-KG) and ɑ-ketobutyrate (ɑ-KB), as well as the prescription drugs rapamycin and metformin, increase follicle autophagy by affecting mTOR and AMPK signaling, thereby facilitating early entry of hair follicles into anagen [9]. Parodi et al. used an in vitro human hair follicle organ culture model to demonstrate that anti-hair loss products with autophagy- promoting effects could significantly induce autophagy in hair follicle cells and prolong hair follicle anagen [10]. However, the effects of autophagy on the metabolism and function of HFSCs during the hair follicle cycle remain unclear. Understanding the mechanism of autophagy in HFSC metabolism is important for the treatment and prevention of hair loss resulting from HFSC activation failure.

In this study, we measured autophagy in HFSC during different hair follicle cycle stages and investigated its effects on the hair follicle cycle and HFSCs. We also investigated the effects and mechanisms of autophagy on HFSC metabolism, and whether autophagy affects HFSC function and the hair follicle cycle by regulating cellular metabolism.

## Results

### Enhanced autophagy in HFSC during the telogen-anagen transition

**Fig. 1:**
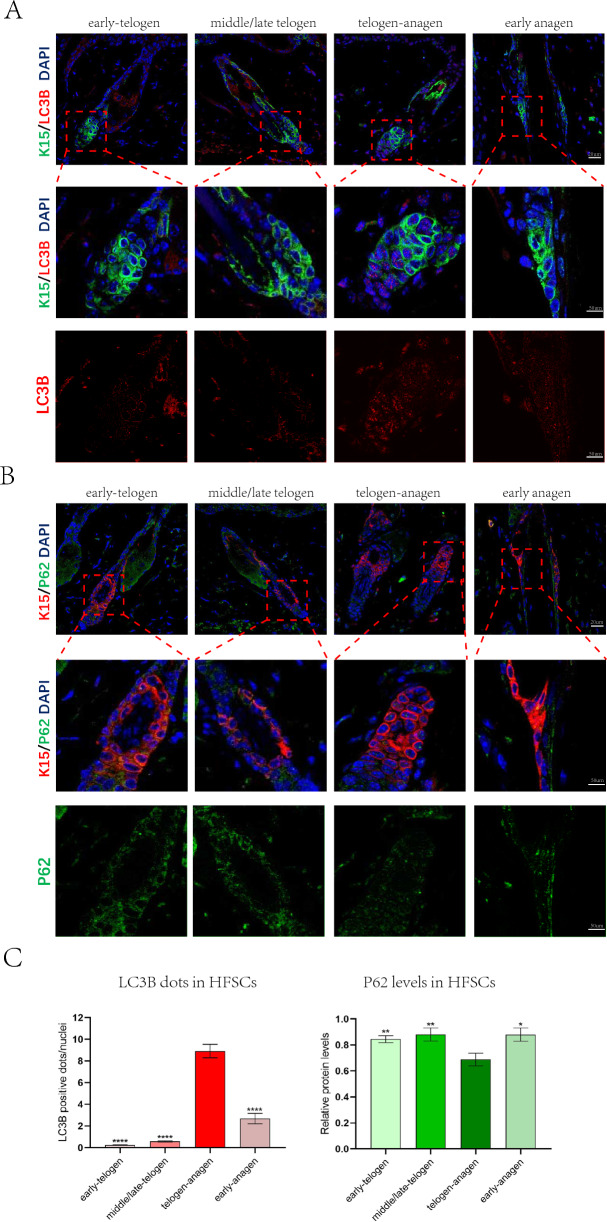
Autophagy is enhanced during the transition from telogen to anagen. **A** Representative immunofluorescence images of dorsal hair follicles stained with anti-LC3B antibody(red) and anti-K15 antibody(green) in early telogen, middle/late telogen, telogen-anagen transition and anagen. **B** Representative immunofluorescence images of dorsal hair follicles stained with anti-P62 antibody(green) and anti-K15 antibody(red) in early telogen, middle/late telogen, telogen-anagen transition and anagen. **C** Quantification of LC3B-fluorescent dots/cell in K15 + HFSCs region of dorsal hair follicles(left); Quantification of P62-fluorescent signal in K15 + HFSCs region of dorsal hair follicles(right). The data represent the means ± S.E.M from at least three independent experiments. *P < 0.05, **P < 0.01, ****P < 0.0001, determined by Student’s t-test, the other stages of hair follicle cycle versus telogen-anagen

There is a significant difference in the cellular activity of telogen and anagen hair follicles. During the telogen, HFSCs remain quiescent until they are activated and proliferate upon receiving growth stimulation signals (Additional file 2: Fig. S1). To detect autophagy in HFSCs during different hair follicle cycle stages, we performed co-localization assays of the autophagy markers LC3B, P62, and HFSC-specific marker K15 on the dorsal skin of mice in the early telogen (50 days after birth), middle/late telogen (63 days after birth), telogen-anagen transition (77 days after birth), and anagen (84 days after birth). Fewer LC3B-positive dots were observed in HFSCs during the early and middle/late telogen; however, the number of LC3B-positive dots briefly increased during the anagen-telogen transition and then decreased after entering the anagen. In contrast, P62 levels decreased during the anagen-telogen transition, which corresponded to the LC3B-positive dots (Fig. 1A–C). Autophagy in HFSCs was induced during the telogen-anagen transition and returned to lower levels after entering the anagen. The alterations of autophagy in HFSCs were consistent with the transition of hair follicle cycle, suggesting that autophagy may play an important role in the transition of hair follicles from telogen to anagen.

### Autophagy regulates the hair follicle cycle and HFSC activation

**Fig. 2:**
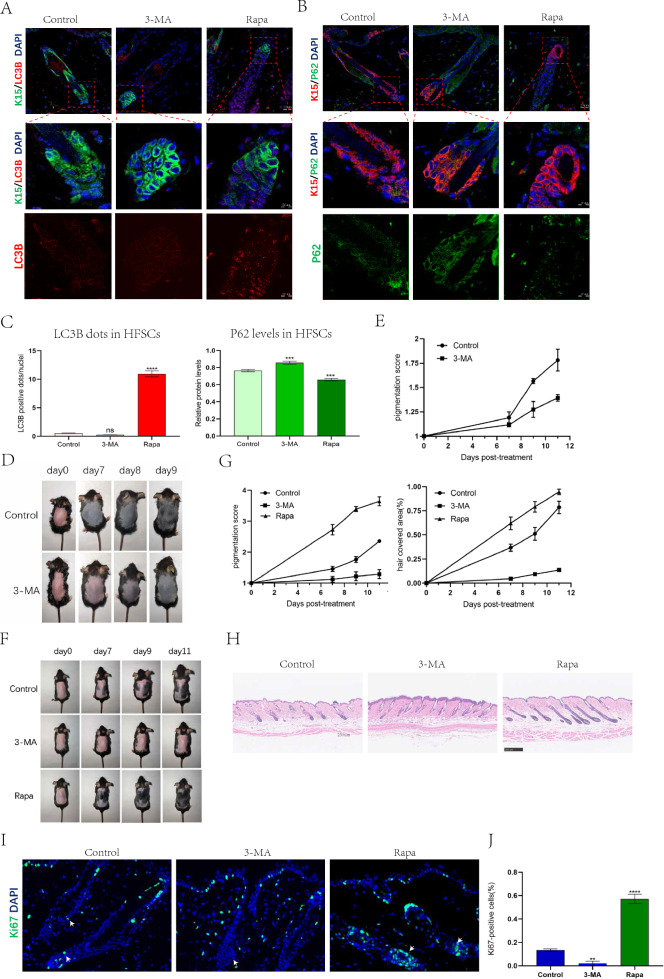
Autophagy promotes mice hair follicle cycle and HFSC activation. **A** Representative immunofluorescence images of dorsal hair follicles treated with control,3-MA (24 h) or Rapa (24 h) stained with anti-LC3B antibody(red) and anti-K15 antibody(green). **B** Representative immunofluorescence images of dorsal hair follicles treated with control,3-MA (24 h) or Rapa (24 h) stained with anti-P62 antibody (green) and anti-K15 antibody(red). **C** Quantification of LC3B-fluorescent dots/cell in K15 + HFSCs region of control,3-MA-treated or Rapa-treated hair follicles(left); Quantification of P62-fluorescent signal in K15 + HFSCs region of control,3-MA-treated or Rapa-treated hair follicles(right). **D** Inhibition of autophagy inhibits hair regeneration.C57BL/6 mice were shaved on postnatal week 8–9 and topically treated with control or 3-MA every other day. Photographs were taken on day 7,9,11 post-treatment. (**E**) Quantification of melanin pigmentation in dorsal skin. **F** Enhanced autophagy induces hair regeneration.C57BL/6 mice were shaved on postnatal week 8–9 and topically treated with control, 3-MA or Rapa every other day. Photographs were taken on day 7,9,11 post-treatment. **G** Quantification of melanin pigmentation and hair covered area on dorsal skin. **H** Microphotographs of H&E stained skin tissue section from mice treated with control, 3-MA or Rapa. **I**, **J** Rapa increases the number of Ki67 + HFSCs (white arrow). **I** Representative immunofluorescence images of dorsal hair follicles treated with control, 3-MA or Rapa stained with anti-Ki67 antibody (green). **J** Quantification for the number of Ki67 + HFSCs in dorsal hair follicle. The data represent the means ± S.E.M from at least three independent experiments. **P < 0.01, ***P < 0.001, ****P < 0.0001, determined by Student’s t-test, 3-MA or Rapa versus Control

To investigate the effect of upregulated-autophagy during the telogen-anagen transition on the hair follicle cycle, we treated mice with drug injection in the mid-late telogen (9–10 weeks after birth). Immunofluorescence staining of mouse HFSCs after treatment for 1 day showed that the number of LC3B-positive dots in the 3-MA group was lower than that in the control group. In contrast, the Rapa group showed a significant increase in LC3B-positive dots. Additionally, the 3-MA group exhibited increased p62 levels in HFSCs, while the Rapa group showed decreased p62 levels. These findings demonstrated that 3-MA inhibited autophagy and Rapa promoted HFSCs autophagy effectively. (Fig. 2A–C). First, we explored the effect of 3-MA alone on the hair follicle cycle by photographing on days 0, 7, 9, and 11 after drug treatment and analyzed the hair growth using imaging software. On day 7, we observed melanosis in the 3-MA and control groups; however, the mice in the 3-MA group had a much lighter skin color compared to the control group. On days 9 and 11, the melanosis rates in the control group were 1.57 and 1.78 times those of the telogen period, while the melanosis rates in the 3-MA group were only 1.29 and 1.42 times those of the telogen period (Fig. 2D, E). This indicated that inhibiting autophagy delayed the telogen-anagen transition in mouse hair follicles. We further investigated whether autophagy promotes the early entry of hair follicles into the anagen in mice. Surprisingly, on day 11, mice in the Rapa group regained almost intact hair, whereas most hair follicles in the 3-MA group remained in the telogen (Fig. 2F, G). Hematoxylin and eosin (H&E) staining of the skin confirmed these findings (Fig. 2H). To investigate whether autophagy had an activating effect on HFSCs, we performed Ki67 staining of mouse hair follicles after treatment for 7 days. On day 7, more HFSCs of the Rapa group showed proliferation, while fewer HFSCs in the control group and almost none in the 3-MA group showed proliferation (Fig. 2I, J). Previous studies have reported that plucking hair follicles from the dorsal skin of mice can unify the hair follicle cycle and induce the early anagen [11]. On the first day after hair plucking, we observed an increase in the number of LC3B-positive dots in HFSCs. To further investigate whether the increase in LC3B-positive dots after plucking is attributed to lysosomal dysfunction, we administered chloroquine (CQ) treatment after plucking. CQ inhibits lysosomal function and induces accumulation of autolysosomes encapsulated by LC3B. We found that there were more LC3B-positive dots in the CQ-treated group compared to the group that underwent hair plucking alone, indicating an active autophagic flux in HFSCs (Additional file 3: Fig. S2) and suggesting that enhanced HFSCs autophagy may be one of the mechanisms for the early entry of hair follicles into anagen after hair plucking.

**Fig. 3:**
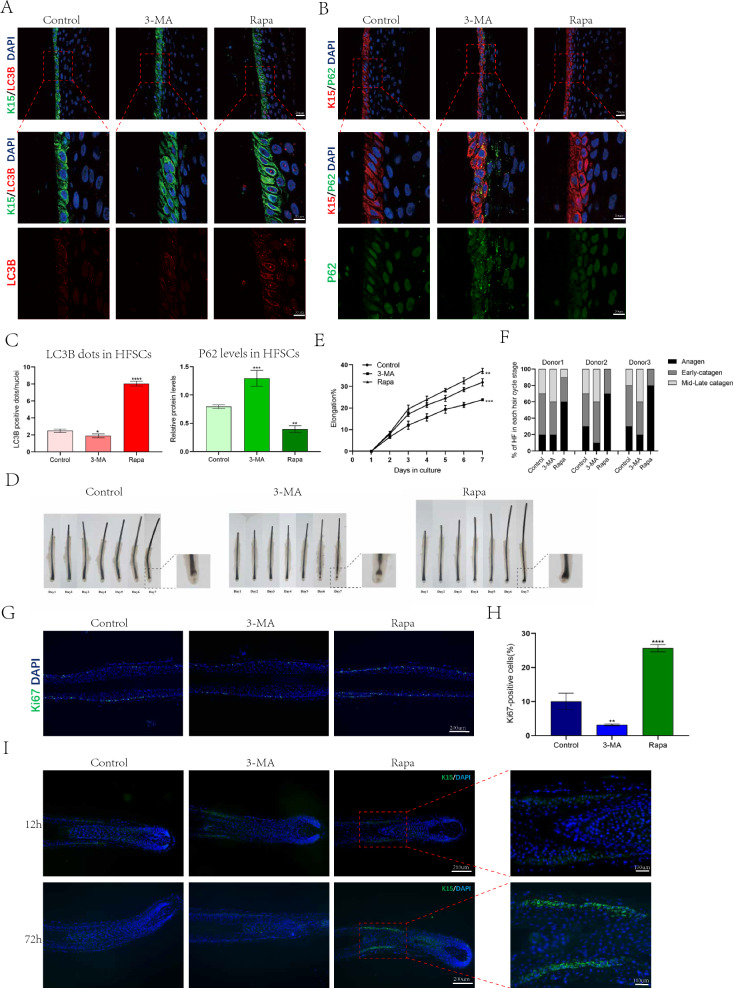
Autophagy promotes human hair follicle cycle and HFSC activation. **A** Representative immunofluorescence images of human hair follicles treated with control,3-MA(8 h) or Rapa(8 h) stained with anti-LC3B antibody(red) and anti-K15 antibody(green). **B** Representative immunofluorescence images of hair follicles treated with control,3-MA(8 h) or Rapa(8 h) stained with anti-P62 antibody(green) and anti-K15 antibody(red). **C** Quantification of LC3B-fluorescent dots/cell in K15 + HFSCs region of control, 3-MA-treated or Rapa-treated hair follicles(left); Quantification of P62-fluorescent signal in K15 + HFSCs region of control,3-MA-treated or Rapa-treated hair follicles(right). **D** Human hair follicles were treated with control,3-MA or Rapa. Photographs were taken for 7 consecutive days. **E** Increased hair shaft elongation after Rapa treatment. Independent experiments were repeated at least three times and data were based on 30 hair follicles from three donors. **F** Recording of hair follicle cycle stages treated with control,3-MA or Rapa on day 7. **G**, **H** Rapa promotes HFSC activation. **G** The proliferative HFSCs were assessed by the proliferative marker, Ki67 (green) on post-treatment day 3. **H** Quantification of Ki67 + cell numbers in control,3-MA-treated or Rapa-treated hair follicles. **I** Representative immunofluorescence images of human hair follicles treated with control,3-MA or Rapa stained with anti-K15 antibody(green) on 12 h and 72 h post-treatment. The data represent the means ± S.E.M. from at least three independent experiments. *P < 0.05, **P < 0.01, ***P < 0.001, ****P < 0.0001, determined by Student’s t-test, 3-MA or Rapa versus Control

To investigate whether autophagy plays a role in regulating the cycle and activating HFSCs in human hair follicles, we conducted an experiment using organ culture of human hair follicles. This allowed us to observe the effect of autophagy on hair follicles under conditions that closely resembled the physiological microenvironment and tissue morphology. Human hair follicles were treated with Rapa (1 nM) and 3-MA (5 uM) for 6 h. We observed a decrease in the number of LC3B-positive dots and an increase in p62 levels in HFSCs treated with 3-MA. In contrast, the Rapa group showed an increase in the number of LC3B-positive dots and a decrease in p62 levels. These findings demonstrated that the drug treatment effectively modulated autophagy in HFSCs (Fig. 3A–C). Next, we examined the morphological changes in the length of each hair follicle after 1–7 days of drug treatment and assessed the hair follicle cycle stages on day 7. We observed that 3-MA retarded hair shaft growth, while Rapa significantly promoted it (Fig. 3D, E). On day 7, the control group had 30% hair follicles in anagen, whereas the 3-MA group had < 20% hair follicles in anagen. In contrast, over 50% of the hair follicles cultured with Rapa remained in anagen (Fig. 3F). To further confirm the impact of autophagy on HFSC, we compared the proportion of proliferating HFSCs and the expression of the HFSC marker K15 after 3 days of culture under different treatments. Rapa treatment significantly increased the percentage of Ki67 + cells in HFSC and their progeny, while 3-MA treatment decreased it compared to those in the control group (Fig. 3G, H). Drug treatment did not seem to affect HFSC K15 expression in the bulge zone. However, Rapa significantly enhanced K15 expression in HFSC progenitor cells above the hair follicle bulb (Fig. 3I). As K15 is a stem cell-specific marker in the bulge zone, these results indicate that at least some progenitor cells regained their stem cell properties.

In summary, upregulated-autophagy activates HFSCs and promotes the initiation and prolongation of anagen, whereas impaired autophagy disrupts in the hair follicle cycle in both mice and humans. Our results support the hypothesis that autophagy plays a critical role in HFSC activation and hair follicle cycle regulation (Additional file 1).

### Induction of autophagy promotes a shift in HFSC metabolism toward glycolysis

Previous studies have shown that autophagy contributes to the maintenance of glycolytic metabolism in hematopoietic stem cells and that impaired autophagy leads to abnormal maintenance and function of hematopoietic stem cells [12]. We hypothesized that autophagy would affect the metabolism of HFSCs. HFSCs are located in the bulge zone of the hair follicle, which is situated beneath the opening of the sebaceous gland and is regarded as a rich niche for HFSCs (Additional file 4: Fig. S3A, B). Compared to its activity in other cells within the hair follicle, K15 promoter activity is considered an exclusive characteristic of HFSCs (Additional file 4: Fig. S3C). To investigate the effect of autophagy on HFSC metabolism, we first examined the effect of different drug concentrations on the percentage of live cells and cell viability (%) using live/dead staining and Cell Counting Kit-8 reagent. HFSCs were extracted as previously described [13] and assayed when cell growth reached a confluence of 80% in dish. The extracted HFSCs exhibited a morphology resembling that of keratinocytes and a cobblestone-like arrangement after 5 days of culture (Additional file 4: Fig. S3D). Immunofluorescence staining confirmed the presence of stem cell marker K15 in extracted cells (Additional file 3: Fig. S3E). After 24 h of drug treatment, there were no discernible changes in the cellular morphology between the control and experimental groups (Additional file 5: Fig. S4A), and the percentage of live cells showed no significant differences (Additional file 5: Fig. S4B). However, 3-MA inhibited cell proliferation, whereas Rapa promoted cell proliferation in a concentration-dependent manner. At concentrations of 0.05 and 2.5 nM, Rapa had no effect on cell proliferation, while 0.5 nM Rapa exhibited the most significant promotion of cell proliferation (Additional file 5: Fig. S4C).

**Fig. 4:**
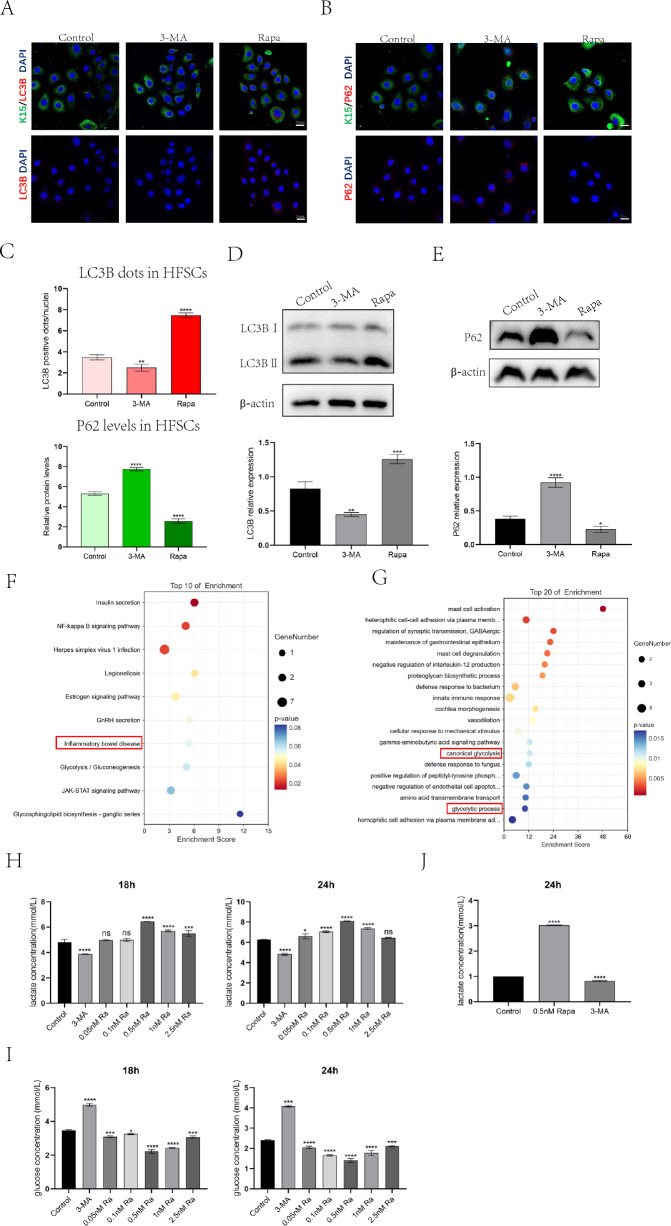
Autophagy induces HFSC glycolysis. **A** Representative immunofluorescence images of HFSCs treated with control, 3-MA(6 h) or Rapa(6 h) stained with anti-LC3B antibody(red) and anti-K15 antibody(green). **B** Representative immunofluorescence images of HFSCs treated with control,3-MA(6 h) or Rapa(6 h) stained with anti-P62 antibody(red) and anti-K15 antibody(green). **C** Quantification of LC3B-fluorescent dots/cell of control, 3-MA-treated or Rapa-treated HFSCs(up); Quantification of P62-fluorescent signal of control, 3-MA-treated or Rapa-treated HFSCs(down). **D**, **E** Protein extracted from HFSCs treated with Rapa (0.5 nM) for 6 h or treated with 3-MA (5 uM) for 24 h. Western blotting analysis of LC3B and P62 expression levels. (**D**) LC3B dots was evaluated 6 h post-administration with control, 3-MA or Rapa. **E** P62 expression was evaluated 24 h post-administration with control, 3-MA or Rapa. β-Actin was used as the loading control. **F** KEGG pathway enrichment analysis bubble chart. **G** Biological process category enrichment bubble chart of GO analysis. The Y-axis of the bubble chart represents GO or KEGG pathway terms, and the color of the bubble represents the P value of the terms. **H**, **I** Analysis of the concentrations of glucose and lactate in HFSCs supernatant cultured with control, 3-MA or different concentrations of Rapa for 18 h and 24 h. **J** Analysis of the concentration of lactate in HFSCs cultured with control, 3-MA or Rapa (0.5 nM) for 24 h. The data represent the means ± S.E.M. from at least three independent experiments. *P < 0.05, **P < 0.01, ***P < 0.001, ****P < 0.0001, determined by Student’s t-test, 3-MA or different concentrations of Rapa versus Control

We also examined autophagy in HFSCs after treatment with Rapa (0.5 nM) or 3-MA (5 mM) for 6 h. Consistent with the results in presented Figs. 2A, B and 3A, B, we observed an increase in LC3B-positive dots and weakened P62 expression in the Rapa group, and a decrease in LC3B-positive dots and enhanced P62 expression in the 3-MA group, which validated the efficacy of the pharmacological intervention (Fig. 4A–C), the results of western blot were consistent with those of the immunofluorescence staining (Fig. 4D, E). These results indicated that the autophagic flux was active in Rapa-treated HFSCs. Next, we performed transcriptome sequencing of the HFSCs treated with Rapa for 24 h. Both KEGG and GO enrichment pathway analyses revealed significant changes in glycolytic metabolism in the top-enriched pathways (Fig. 4F, G). To investigate the relationship between autophagy and HFSC glycolysis, we collected culture supernatants from HFSCs treated with 3-MA and different concentrations of Rapa for 18 h and 24 h, and measured the lactate and glucose concentrations in the supernatants. The results showed that Rapa promoted glucose consumption and lactate production in HFSCs, in which 0.5 nM Rapa had the most significant and rapid effect on HFSCs. In contrast, 3-MA significantly inhibited glucose consumption and reduced lactate production in the HFSCs (Fig. 4H, I). We also measured the intracellular lactate concentration and found that the lactate concentration in HFSCs after Rapa treatment was 3.75 times that after 3-MA treatment (Fig. 4J). These results suggest that the induction of autophagy can promote a shift in HFSC metabolism toward glycolysis and increase glucose consumption and lactate production in HFSCs.

### Autophagy promotes Ldha expression and up-regulates Ldha activity in HFSCs

**Fig. 5:**
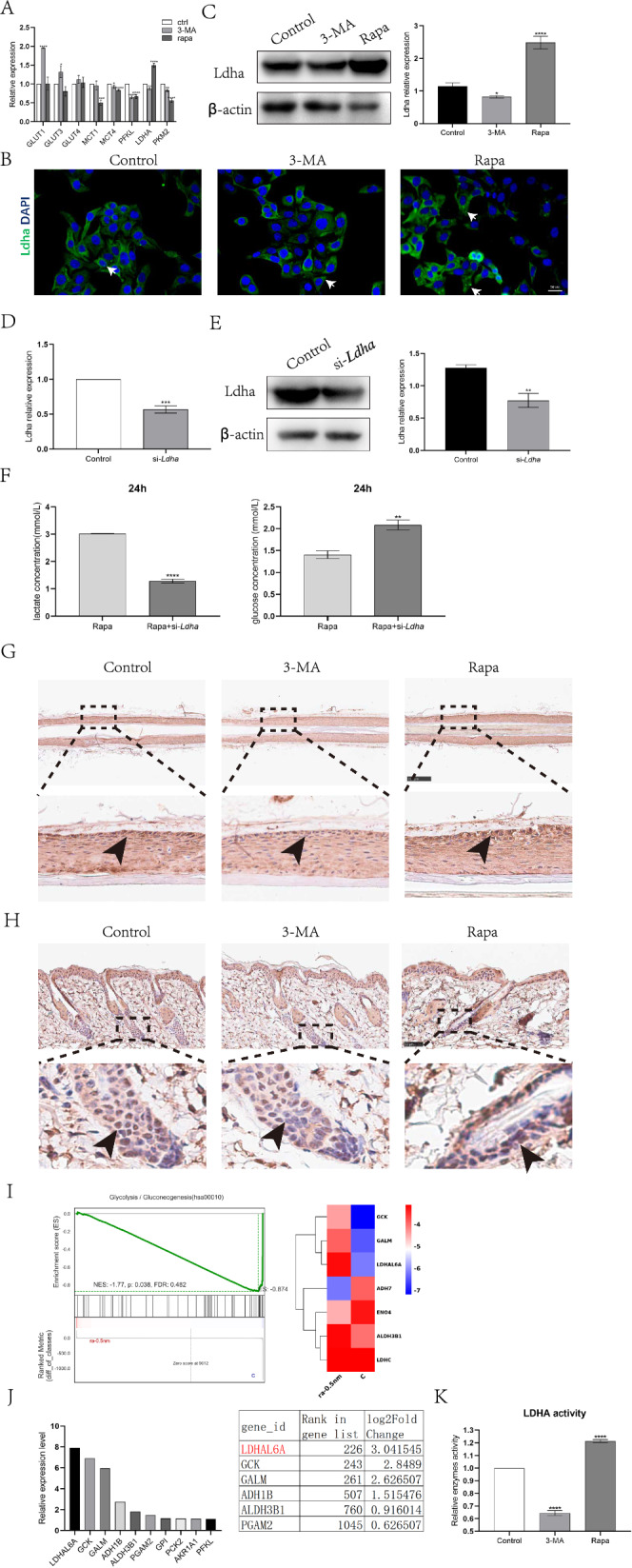
Autophagy induces HFSC glycolysis by upregulating Ldha expression and increasing Ldha activity. **A**–**C** The expression levels of glycolysis key enzymes in HFSCs cultured with control, 3-MA or Rapa for 24 h were detected by RT-qPCR, immunofluorescence staining and western blot, p value was conducted by 3-MA or Rapa versus Control. **D**, **E** RT-qPCR and western blot were used to detect the expression of Ldha in HFSCs transfected with Ldha siRNA, p value was conducted by Rapa versus Control. **F** Analysis of the concentrations of glucose and lactate in HFSCs supernatant cultured with Rapa or Rapa + si-*Ldha* for 24 h. P value was conducted by Rapa + si-*Ldha* versus Rapa. **G**, **H** Rapa promotes Ldha expression in vitro and in vivo (black arrow represents the location of HFSCs). **G** Representative immunohistochemical images of human hair follicles treated with control, 3-MA (24 h) or Rapa (24 h) stained with anti-Ldha antibody. **H** Representative immunohistochemical images of dorsal hair follicles treated with control, 3-MA(24 h) or Rapa(24 h) stained with anti-Ldha antibody. **I** Gene set enrichment analysis (GSEA) of RNA-seq transcriptome data from HFSCs treated with control or Rapa shows enrichment for glycolysis/gluconeogenesis pathway (left) and the gene signature (right), with LDHAL6A as the top up-regulated gene. **J** Fold change of TE for glycolytic genes between Rapa-treated HFSCs with control HFSCs. **K** Relative Ldha activity in HFSCs treated with control, 3-MA (24 h) or Rapa (24 h), p value was conducted by 3-MA or Rapa versus Control. The data represent the means ± S.E.M from at least three independent experiments. *P < 0.05, **P < 0.01, ***P < 0.001, ****P < 0.0001, determined by Student’s t-test

Previous studies have confirmed that autophagy regulates cellular glycolysis by modulating the expression of glycolytic metabolic enzymes [13–15]. Therefore, to investigate the regulatory mechanisms of autophagy in HFSC metabolism, we examined the expression of key glycolytic enzymes in HFSCs using RT-qPCR. We found that Rapa upregulated Ldha expression in HFSCs, whereas 3-MA inhibited it (Fig. 5A–C). To investigate the role of Ldha in autophagy-induced HFSC glycolysis, we transfected HFSCs with *Ldha* siRNA for 48 h to reduce Ldha expression. The transfection efficiency of si-*Ldha* was detected by RT-qPCR and western blotting, and the results indicated that si-*Ldha* effectively reduced the expression of Ldha (Fig. 5D, E), consistent with the findings of a previous study [15]. *Ldha* knockdown reduced the rate of lactate production and glucose consumption in HFSCs treated with Rapa (Fig. 5F). These results indicate that autophagy regulates glycolysis in HFSCs by promoting the expression of Ldha.

We then verified the role of autophagy in promoting Ldha expression in human hair follicles. We treated human hair follicles with Rapa or 3-MA for 24 h, then, detected Ldha expression by immunohistochemical staining. Rapa significantly increased human hair follicle Ldha expression in HFSCs, whereas 3-MA inhibited it (Fig. 5G). We injected drugs or solvent into the dorsal skin of C57 mice and performed assays after 24 h. Consistent with the results of the human hair follicle assay, Rapa promoted Ldha expression in mouse HFSCs, whereas 3-MA inhibited it (Fig. 5H). In addition, differential gene expression analysis of the glycolytic pathway revealed that LDHAL6A was the glycolytic enzyme with the most significant changes in Rapa-treated HFSCs (Fig. 5I, J). As LDHAL6A is considered to be positively correlated with Ldha activity, we lysed HFSCs and subjected the cell lysates to an enzymatic assay similar to the colorimetric assay. The results showed that Rapa significantly increased Ldha activity in HFSCs (Fig. 5K). These results suggest that autophagy shifts HFSC metabolism toward glycolysis by promoting Ldha expression and increasing Ldha activity in HFSCs.

### Autophagy activates HFSC and induces hair growth by promoting glycolysis

**Fig. 6:**
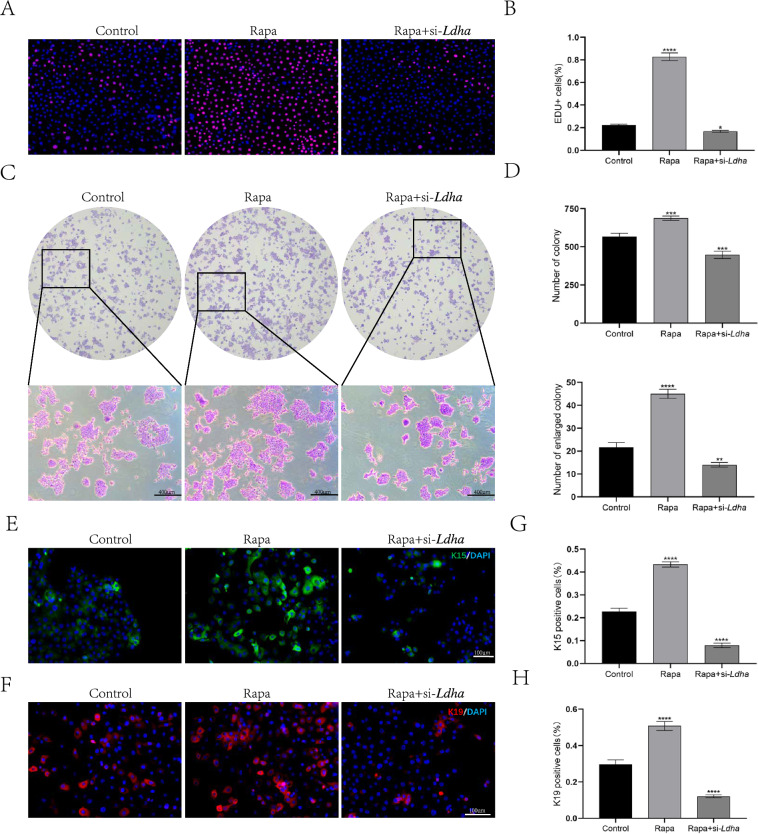
Autophagy-induced glycolysis activates HFSC and preserves their stemness. **A** HFSCs treated with control, Rapa or Rapa + si-*Ldha* were subjected to the EdU labeling assay (EdU-positive(red); Hoechst(blue)). **B** Quantification of the proportion of EdU-positive cells in control, Rapa-treated or Rapa + si-*Ldha*-treated group. **C** Colony formation assays further confirmed that Rapa increased the proliferation of HFSCs and si-*Ldha* impaired Rapa-induced proliferation. **D** Quantification of the number of colony (up) and enlarged colony (> 100 cells/colony, down). **E**, **F** Immunofluorescence staining with anti-K15 antibody or anti-K19 antibody of HFSCs treated with control, Rapa or Rapa + si-*Ldha* was performed on day 3 of culturing. **G**, **H** Quantification of K15 + cells or K19 + cells numbers in control, Rapa-treated or Rapa + si-*Ldha* group. The data represent the means ± S.E.M from at least three independent experiments. *P < 0.05, **P < 0.01, ***P < 0.001, ****P < 0.0001, determined by Student’s t-test, Rapa or Rapa + si-*Ldha* versus Control

To further investigate the effect of autophagy-induced Ldha expression on HFSCs and hair follicle cycle, we performed EdU staining of HFSCs and found that Rapa promoted HFSCs proliferation, whereas transfection with si–*Ldha* notably diminished the promotive effect of Rapa on HFSCs' proliferation (Fig. 6A, B). We used a clone formation assay to evaluate the effects of autophagy on the self-renewal capability of HFSCs. The results showed that Rapa significantly increased the number of HFSC colonies and the cell count within each individual HFSC colony, while transfection with si-*Ldha* inhibited the promoting effect of Rapa on HFSC colony formation (Fig. 6C, D). To investigate whether autophagy excessively activates HFSCs and leads to the loss of their stem cell properties, we measured the number of K15 + and K19 + HFSCs by immunofluorescence staining. Rapa significantly increased the number of K15 + and K19 + HFSCs (Fig. 6E, F), with K15 + HFSCs being twice that of the control group and 5.25 times that of the Rapa + si-*Ldha* group. Meanwhile, K19 + HFSCs were 1.6 times that of the control group and 4 times that of the Rapa + si-*Ldha* group (Fig. 6G, H). These results suggest that autophagy activates HFSCs by upregulating Ldha expression and maintaining their self-renewal capability, thus preserving stem cell properties within at least a portion of the HFSCs.

**Fig. 7:**
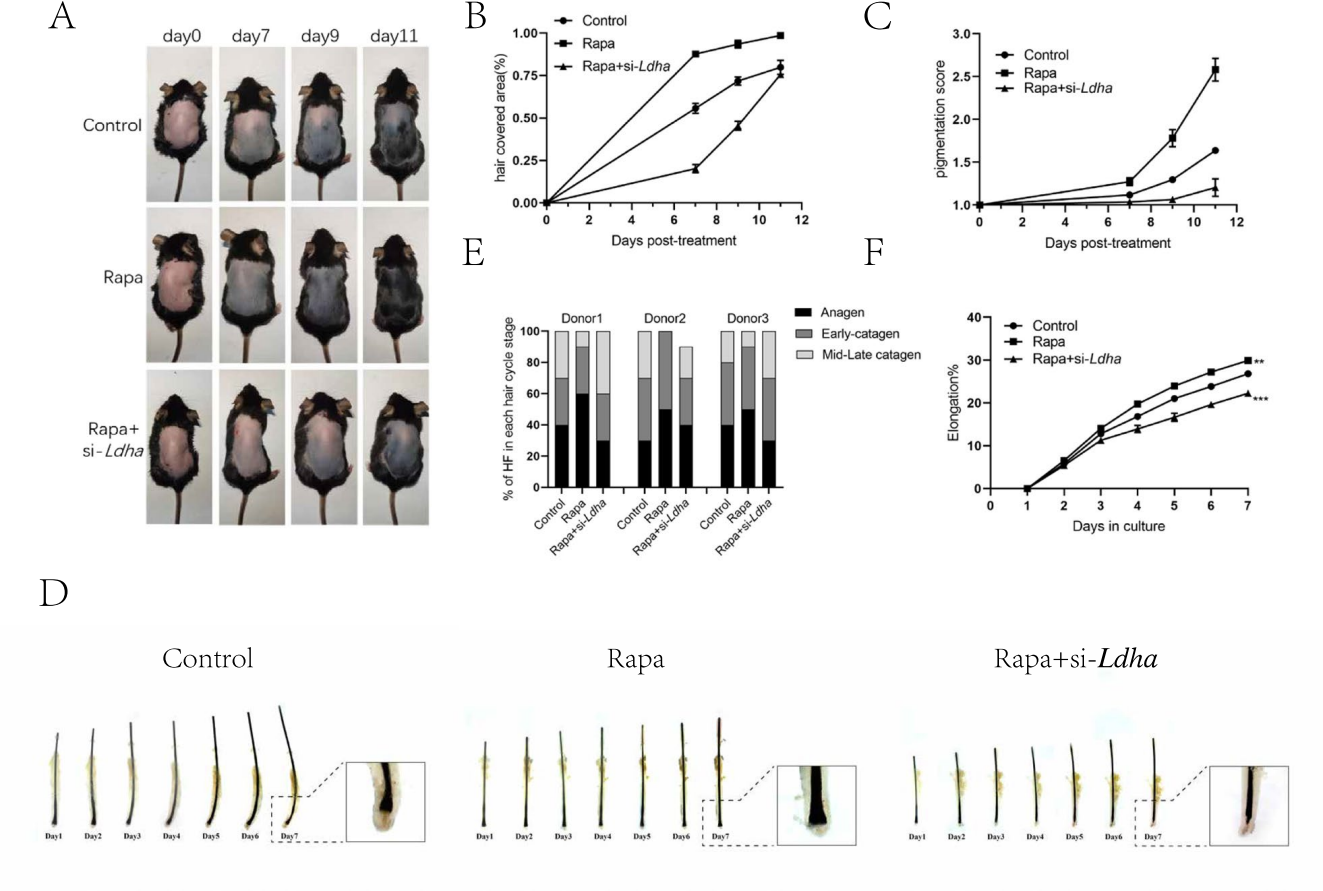
Autophagy-induced glycolysis regulates the hair follicle cycle. **A** C57BL/6 mice were shaved on postnatal week 8–9 and topically treated with control, Rapa or Rapa + si-*Ldha* every other day. Photographs were taken on day 7,9,11 post-treatment. **B**, **C** Quantification for melanin pigmentation and hair covered area on dorsal skin. **D** Human hair follicles were treated with control, Rapa or Rapa + si-Ldha. Photographs were taken for 7 consecutive days. **E**, **F** Quantification of hair follicle cycle stages and hair shaft elongation of hair follicles treated with control, Rapa or Rapa + si-Ldha

Finally, to investigate whether autophagy-induced Ldha expression is a key factor in altering the hair follicle cycle, we injected Rapa solution, Rapa + si-*Ldha* solution or solvent into the dorsal skin of C57 mice every other day and recorded the hair follicle coverage area (%) on days 0, 7, 9, and 11. On day 11, hair follicles in Rapa-treated mice mostly entered anagen, whereas most hair follicles in Rapa + si-*Ldha-*treated mice were still in the telogen-anagen transition. This indicated that transfection with si-*Ldha* significantly inhibited Rapa-induced hair follicle growth (Fig. 7A–C). Human hair follicles were cultured in William's E conditioned medium containing Rapa solution, Rapa + si-Ldha solution or solvent, and the length changes of each hair follicle after 1–7 days of treatment and the stage of hair follicle cycle at day 7 were assessed. On day 7, compared to hair follicles treated with Rapa alone, the Rapa + si-*Ldha* group showed a 10–30% decrease in anagen and a 20–30% increase in catagen, and the elongation rate also decreased by approximately 10% (Fig. 7D–F). These results indicated that transfection with si-*Ldha* significantly inhibited the effects of Rapa on prolonging hair follicles anagen and promoting hair follicle growth.

In summary, these data suggest that autophagy activates HFSC by upregulating Ldha expression to promote the shift of HFSC metabolism toward glycolysis, thereby regulating the hair follicle cycle and promoting hair growth.

## Discussion

HFSCs are adult stem cells with the ability to self-renew and differentiate into various cell types. During the anagen, HFSCs are activated and begin to proliferate and differentiate to replenish the epidermal components of hair follicles, such as hair matrix cells. During the telogen, they maintain quiescence until the next hair follicle cycle begins. In pathological states, such as AGA, impaired activation of HFSCs results in shortened anagen and extended telogen, causing a hair loss phenotype. Therefore, understanding the mechanism of regulating HFSC activation is of great significance for the treatment of hair loss diseases.

Cellular autophagy maintains the metabolic homeostasis of cells and regulates stem cell activation by degrading soluble macromolecules and denatured organelles in the cytoplasm [16]. Autophagy in mouse dorsal skin remains low during the telogen and increases upon entering the anagen. The Inhibition of autophagy causes hair follicles to enter the catagen earlier [9, 10], highlighting the importance of autophagy in initiating the normal hair follicle cycle and hair regeneration under physiological conditions. In a mouse model of alopecia areata, researchers found that autophagy within hair follicles was suppressed, and the induction of autophagy alleviated alopecia symptoms, whereas its inhibition advanced disease development [17]. Parodi et al. showed that hair matrix cells could potentially serve as target cells for autophagy-mediated regulation of the hair follicle cycle [10]. However, owing to the rapid division and apoptosis of hair matrix cells, there are still significant limitations in treating hair loss by regulating these cells. In contrast, as mother cells of hair matrix cells and seed cells for hair follicle regeneration, HFSCs persist in hair follicles for a long period. This raises question of whether autophagy, an essential physiological process in cells that responds to external stimuli and maintains cellular functions, also regulates the function of HFSCs? To our knowledge, this is the first study to report changes in autophagy in HFSCs at different stages of the hair follicle cycle. We also explored the effects of autophagy on HFSC activation and its potential mechanisms. Autophagy in HFSC remains low during telogen and anagen and increases significantly during the telogen-anagen transition, which is highly consistent with the hair follicle cycle. This suggests that autophagy may play a crucial role in promoting HFSC activation, thereby initiating the hair follicle cycle (Fig. 1). To further clarify the regulatory effects of autophagy on HFSC activation, 3-MA and Rapa were used to pharmacologically modulate autophagy. The data showed that under physiological conditions, inhibition of autophagy impeded the hair follicle cycle, whereas stimulation of autophagy activates HFSC, promotes HFSC proliferation and ultimately advances the hair follicle anagen (Fig. 2). Furthermore, we used the human hair follicle organ culture model to investigate whether autophagy-mediated regulatory effects on HFSC activation and the hair follicle cycle are conserved in humans. Consistent with the results in mice, we found that the induction of autophagy promoted human HFSC proliferation and prolonged anagen of hair follicles. Unexpectedly, we also found that the induction of autophagy restored the stem cell properties of HFSC (Fig. 3). Although we observed that autophagy is effective in activating and restoring the stem cell properties of some HFSC progenitors, we did not explore whether autophagy plays different roles in HFSCs and their progenitors, which requires further researches.

Metabolism plays a crucial role in autophagy-regulated functions of stem cells [18]. Current research indicates that glycolysis, lipid metabolism and glutamine metabolism can affect HFSC activation in hair follicles [5, 19]. However, it remains unclear whether autophagy activates HFSC by regulating metabolism, or which specific type of metabolism is involved. To further explore the mechanism by which autophagy regulates HFSC activation, we conducted transcriptome sequencing and found that autophagy may be associated with glycolysis. Further experiments confirmed that autophagy could regulate HFSC glycolysis (Figs. 4 and 5) by upregulating Ldha expression and activity, and promoting lactate production in HFSCs. Flores et al. suggested that HFSCs utilize glycolytic metabolism more than other skin epidermal cells, and that the increased production of lactate seems crucial for HFSC activation [5]. By knocking out *Ldha*, we confirmed the effect of upregulated-glycolysis on autophagy-mediated HFSC activation and the initiation of hair follicle anagen. Transcriptome data analysis revealed that the glycolytic enzyme LDHAL6A may also play a role in autophagy-mediated HFSC activation (Fig. 5G, H). However, the specific mechanisms by which autophagy regulates glycolysis in HFSCs remain to be explored. In addition, because of the involvement of metabolic changes and cell reprogramming in our study, it is necessary to further investigate whether autophagy in HFSCs and their progeny includes other types of autophagy, such as chaperone-mediated autophagy or mitophagy. Furthermore, as autophagy plays a significant role in preventing aging and regulating sebaceous gland function, the effects of autophagy on other constituent cells of hair follicles should be explored in the future [20, 21].

In conclusion, autophagy activates HFSCs and regulates the hair follicle cycle to promote hair regeneration by enhancing Ldha expression and activity in HFSCs, leading to increased lactate production and a shift in HFSCs metabolism toward glycolysis. Moreover, pharmacological interventions targeting autophagy have been shown to promote HFSCs activation and restore the stem cell properties of some progeny cells. These results emphasize that autophagy is a novel target for activating HFSCs and regulating the hair follicle cycle in the treatment of human hair loss.

## Conclusions

Autophagy in HFSCs was the highest during the transition from telogen to anagen. Furthermore, autophagy activates HFSCs by enhancing glycolysis, ultimately initiating the hair follicle cycle and promoting hair follicle regeneration.

## Materials and methods

### Animals

C57BL/6 J female mice (7–8 weeks) were purchased from the Experimental Animal Center of Southern Medical University (Guangzhou, China) and were fed under specific pathogen-free conditions. The mice were shaved on postnatal week 8–9 and randomly assigned to different groups, involving control, 3-MA or Rapa. On postnatal week 9–10, the mice were injected with solvent (0.1% DMSO), 3-MA (5 mM, 50 ul) or Rapa (10 nM, 50ul). The animal experiments were approved by the Animal Research Committee, Southern Medical University (Guangzhou, China). All mice studies were conducted under the instructions and permissions of the Animal Care and Use Committee at the International Medical Center.

### Immunocytochemistry and Immunohistochemistry

Back skins were harvested from mid-dorsal areas of mice. Then fixed Back skins in 4% formalin solution (Sigma) overnight and dehydrated for embedding in paraffin. 5um paraffin sections were being used in hematoxylin/eosin staining or other staining. Cells were seeded (1 × 10^4^ cells per well) in 24-well plates and cultured until approximately 50% confluency. Then cells were fixed in 4% paraformaldehyde and permeabilized with 0.1% Triton X-100 (Solarbio) in PBS for 10 min at room temperature. Further, the sections or cells were blocked with 3% BSA and incubated with antibodies in 1% BSA for 2 h at room temperature. The antibodies used for IHC or IF included: K15 (1:200, Abcam), K19 (1:200, Abcam), LC3B (1:200, proteintech), P62 (1:200, proteintech), Ldha (1:200, proteintech). The samples were incubated with the following fluorescence-labeled secondary antibodies in the dark for 1 h at room temperature. Cy3 anti-mouse secondary antibodies (1:500, Beyotime, China), Goat Anti-Rabbit lgG AF 594 (1:200, Abmart), Goat Anti-Rabbit lgG AF 488 (1:200, Abmart). The nuclei were stained with DAPI (Life T echnologies, USA) prior to imaging with a confocal laser scanning microscope. For quantification of LC3B positive dots and p62 levels, only HFSCs in the bulge zone (K15 + cells) were counted. Confocal images were taken using a LSM980 Confocal Microscope with standardized light exposure. Images were generated by collecting a stack of 20–30 pictures using 20 × or 40 × objectives. Calculation of LC3B positive dots and P62 protein levels pigmentation scores: the pigmentation score of LC3B was calculated by dividing the number of LC3B positive dots by the number of cell nuclei, and the pigmentation score of P62 was calculated by dividing the P62 protein density by the number of cell nuclei. First, the images were opened in ImageJ/Fiji, and the K15 + HFSCs region was selected, with areas outside this region being removed. The images were then separated into different color channels and converted to 8-bit format. Thresholds for cell nuclei, LC3B, and P62 were subsequently set. The measurement function was used to analyze the number of LC3B-positive dots, the density of P62 protein, and the number of cell nuclei. Finally, the number of LC3B-positive dots per nucleus and P62 protein levels were calculated using the specified formula.

### Hair follicles culture

Hair follicles were cultured in 24-well plates in Williams’E medium supplemented with insulin, hydrocortisone, l-glutamine and Penicillin–Streptomycin solution at 37 °C in a 5% CO_2_ atmosphere. Hair follicles were treated with control, 5 uM 3-MA or 1 nM Rapa every other day. The elongation of each hair follicle was recorded for consecutive 7 days and the hair follicle cycle stage was recorded on day 7 (ten hair follicles were analyzed each experiment). The data were obtained from three independent experiments.

### Culturing of HFSCs

The bulge zone between the isthmus and the upper part of the hair follicle was separated and the HFSCs were extracted and cultured as described before [13]. Briefly, the isolated bulge tissue was treated with 0.1% Dispase for 20 min and the dermis were separated. The hair shift was treated with 0.025% trypsin (Gibco, Gaithersburg, MD, United States) for 5–6 min at 37◦C. The cells suspension was filtered through a 70 µm filter (Corning, Corning, NY, United States) and centrifuged at 1000r for 3 min. Finally, the cells were seeded in six-well plates and cultured in KGM Gold Bulletkit (Lonza, Switzerland) at 37 °C in a 5% CO_2_ atmosphere. Once the cell growth reached a confluence of 80% in dish, cells were harvested with 0.25% trypsin, split at a 1:2 ratio, and maintained in KGM Gold Bulletkit.

### Cytotoxicity assay

HFSCs were cultured in black 96-well plates for 24 h. The culture medium was replaced with 5 uM 3-MA or 0.05, 0.1, 0.5, 1, 2.5 nM Rapa or PBS control in KGM for 24 h. The cytotoxicity of 3-MA and Rapa was examined using the (Calcein/PI Cell Viability/Cytotoxicity Assay Kit (Beyotime, China) according to the manufacturer’s instructions. The percentage of live cells were measured using a microplate reader (Thermo Fisher, America). Five replicates were analyzed each experiment. The data were obtained from three independent experiments.

### Cell proliferation assay

HFSCs treated with 5 uM 3-MA or 0.05,0.1,0.5,1, 2.5 nM Rapa or PBS control in KGM for 24 h, and cell proliferation capability was measured using the Cell Counting Kit-8 (CCK-8; Dojindo Molecular Technologies) according to the manufacturer's protocol (five replicates were analyzed each experiment). The data were obtained from three independent experiments. The effects of control, Rapa and Rapa + si-*Ldha* on HFSCs proliferation were examined using the EdU labeling assay according to the manufacturer's protocol. The cell proliferation capability was examined using fluorescence microscope (IX73 FL, Olympus). Image processing was conducted by ImageJ/Fiji software (three pictures were analyzed each experiment). The data were obtained from three independent experiments.

### Western blotting

Cells proteins were extracted using Cell lysis buffer for Western and IP (Beyotime, Shanghai, China) containing 1% Phenylmethanesulfonyl fluoride (PMSF, Beyotime, Shanghai, China). The protein sample was denaturated at 100 °C for 5 min using sodium dodecyl sulfate–polyacrylamide gel electrophoresis (SDS-PAGE) loading buffer. Proteins of equivalent amounts were distinguished using SDS-PAGE and then transferred to a polyvinylidene fluoride membrane, which was then incubated with primary antibodies against LC3B (1:2000, proteintech), P62 (1:10,000, proteintech), Ldha (1:10,000, proteintech) and β-actin (1:10,000, proteintech) overnight at 4 °C. Enhanced chemiluminescence reagents (Beyotime, Shanghai, China) were used to visualize the immunoreactive bands. Protein band intensity was quantified by ImageJ/Fiji software and normalized to the β-actin loading control.

### RNA-sequencing (RNA-seq)

Total RNA of the control and Rapa-treated (0.5 nM) HFSCs were extracted from three independent experiments together using the Trizol reagent (Invitrogen, CA, USA) according to the manufacturer’s protocol. RNA purity and quantification were evaluated using the NanoDrop 2000 spectrophotometer (Thermo Scientific, USA). RNA integrity was assessed using the Agilent 2100 Bioanalyzer (Agilent Technologies, Santa Clara, CA, USA). Then the libraries were constructed using VAHTS Universal V6 RNA-seq Library Prep Kit according to the manufacturer’s instructions. The libraries were sequenced on llumina Novaseq 6000 platform and 150 bp paired-end reads were generated. About 47.5 raw reads for each sample were generated. Raw reads of fastq format were firstly processed using fastp and the low quality reads were removed to obtain the clean reads. Then about 46.6 clean reads for each sample were retained for subsequent analyses. The clean reads were mapped to the homo sapiens using HISAT2. FPKM of each gene was calculated and the read counts of each gene were obtained by HTSeq-count. Differential expression analysis was performed using the DESeq. P < 0.05 and foldchange > 1.5 or foldchange < 0.67 was set as the threshold for significantly differential expression gene (DEGs). KEGG (Kyoto Encyclopedia of Genes and Genomes) pathway, GO (Gene Ontology) enrichment analysis and GSEA (Gene Set Enrichment Analysis) were performed using oeCloud (cloud.oebiotech.com).

### Lactate level and glucose measurement

Cells supernatant lactate concentrations were detected using a Lactate Assay Kit (Nanjing Jiancheng, China) and glucose concentrations were detected using GLU Assay Kit (Nanjing Jiancheng, China) according to the manufacturer's protocol. The cells supernatant of HFSCs was taken at 18 h and 24 h after treated with control,5 uM 3-MA or 0.05, 0.1, 0.5, 1, 2.5 nM Rapa. Cellular inner lactate concentrations were detected using a Lactate Concentration Assay Kit (Solarbio, China) according to the manufacturer's protocol. The data were obtained from three independent experiments.

### Quantitative real-time polymerase

Total RNA was extracted from the cells treated with control,5uM 3-MA or 0.5 nM Rapa using Trizol (Invitrogen, CA, USA). The mRNA was reverse-transcribed to cDNA using the Tissue RNA Purification Kit Plus (EZBioscience, America) according to the manufacturer’s protocol. The qRT-PCR analysis was performed with Hieff® qPCR SYBR Green Master Mix (YEASEN, China) in a Light Cycle Roche 480 II Real-time PCR system (Roche, Basel Switzerland). Three replicates were analyzed. The primer sequences used for qRT-PCR analysis are listed in Table 1. The expression levels of target genes were normalized with β-actin (internal control).

**Table 1:** Primer sequences for qRT-PCR

Gene	Forward primer (5'-3')	Reverse primer (5'-3')
β-actin	GGGAAATCGTGCGTGACATTAAG	TGTGTTGGCGTACAGGTCTTTG
GLUT1	CTTTGTGGCCTTCTTTGAAGT	CCACACAGTTGCTCCACAT
GLUT3	ATGCCCTACCAATATCCAGCA	GCTCCCAGTGGACTCATCTG
GLUT4	TGCTCGATTATGCACTGGAAGT	ATGAACCCCATACTCCTTCCCAG
MCT1	AAAGTGGTGAGCTGCGACGTGA	CGTTATATGCGCGGATCGCAG
MCT4	GATATGGGCGCTTACCATTTTCG	TGTGCTGCGTGACATTCCAA
PFKL	AGATGCGCACCAGCATCAACG	GAACCCGGCACATTGTTGGA
LDHA	GGAGGACCCAGCAATTAGTCT	GTTCACCCATCGCGGTTTAT
PKM2	ATGGCTGACACATTCCTGGAGC	CCTTCAACGTCTCCACTGATCG

### Ldha activity measurement

Cells Ldha activity was detected using Ldha Activity Assay Kit (Solarbio, China) according to the manufacturer’s protocol. HFSCs were treated with control, 5 uM 3-MA or 0.5 nM Rapa for 24 h, and 1 × 10^6^ cells were collected for measurement. The data were obtained from three independent experiments.

### Small interfering RNA interference assay

For ex vivo si-*Ldha* experiments, HFSCs were transfected with small interfering RNA (siRNA) targeting human Ldha or a negative control. The si-*Ldha* sequence was as follows: forward 5’-GGAGAAAGCCGUCUUAAUUTT-3’ and reverse 5’-AAUUAAGACGGCUUUCUCCTT-3’, which was designed and synthesized by IGE BIOTECHNOLOGY (Guangzhou, China). For in vivo si-*Ldha* experiments, C57BL/6 mice were shaved on postnatal week 8–9 and were randomly assigned to different groups. On postnatal week 9–10, mice were treated with si-*Ldha* (10 nM, 50ul) + Rapa (10 nM, 50ul), Rapa (10 nM, 50ul) or control siRNA via direct injection into the dorsal skin every other day. Photographs were taken on day 7,9,11 post-treatment.

### Colony formation assay

The HFSCs (1 × 10^5^ cells per well) seeded in six-well plates were treated with control, Rapa and Rapa + si-*Ldha* for 72 h. The cells were fixed in 4% paraformaldehyde (Solarbio) for 10 min and stained with crystal violet for 10 min. Image processing was using the ImageJ/Fiji software (three pictures were analyzed). The data were obtained from three independent experiments.

### Statistical analysis

All statistical analyses were performed using the GraphPad Prism 8 software. The data were expressed as mean ± S.E.M. All data were analyzed using the Student’s t-test. P < 0.05 was considered significance of difference. All treatments were repeated at least two times.

### Supplementary Information

**Additional file 1. **

**Additional file 2: Fig S1.** Ki67 immunofluorescence staining of hair follicle telogen and anagen. Ki67 expression was significantly upregulated in HFSC (white arrow) during anagen, indicating that the HFSC is active.

**Additional file 3: Fig S2.** **A** Representative immunofluorescence images of dorsal hair follicles stained with anti-LC3B antibody(red) and anti-K15 antibody(green) after plucking hair follicles for 24 h. **B** Representative immunofluorescence images of dorsal hair follicles stained with anti-LC3B antibody(red) and anti-K15 antibody (green) after plucking hair follicles and topically treated with CQ for 24 h.

**Additional file 4: Fig S3.** Characteristics of HFSCs obtained from the bulge of human hair follicles. **A** Morphology of single human hair follicle. **B** Microphotographs of H&E stained human hair follicle. **C** Immunofluorescence images of HFSCs stained with anti-K15 antibody(green) showing the location of bulge zone. **D** Morphology of human HFSCs cultured for 3 days(up) and 5 days(down). **E** Representative immunofluorescence images of human HFSCs stained with anti-K15 antibody(green).

**Additional file 5: Fig S4.** **A** HFSCs phenotypes after cultured with control, 3-MA or Rapa for 5 days. **B** Analysis of live/dead staining of HFSCs cultured with control, 3-MA or different concentrations of Rapa for 24 h. **C** Analysis of CCK8 assay of HFSCs cultured with control, 3-MA or different concentrations of Rapa for 24 h. The data represent the means ± S.E.M from at least three independent experiments. **P < 0.01, ***P < 0.001, ****P < 0.0001, determined by Student’s t-test, 3-MA or different concentrations of Rapa versus Control.

## Data Availability

The datasets generated and analyzed during the current study are available in the Gene Expression Omnibus repository, hyperlink to dataset in https://www.ncbi.nlm.nih.gov/geo/query/acc.cgi?acc=GSE229890.

## Abbreviations

ɑ-KB: ɑ-Ketobutyrate
ɑ-KG: ɑ-Ketoglutarate
3-MA: 3-Methyladenine
AGA: Androgenic alopecia
CK15: Cytokeratin-15
CK19: Cytokeratin-19
CQ: Chloroquine
H&E: Hematoxylin and eosin
HFSCs: Hair follicle stem cells
Ldha: Lactate dehydrogenase
ORS: Outer root sheath
OXPHOS: Oxidative phosphorylation
PCR: Polymerase chain reaction
Rapa: Rapamycin
SGSCs: Salivary gland stem cells
TCA: Tricarboxylic acid
