# Supplementary figures and images for: Autophagy induces hair follicle stem cell activation and hair follicle regeneration by regulating glycolysis
Source: Cell Biosci. 2024 Jan 5;14:6. doi: 10.1186/s13578-023-01177-2 (PMC10770887; doi:10.1186/s13578-023-01177-2)

telogen

Ki67 DAPI

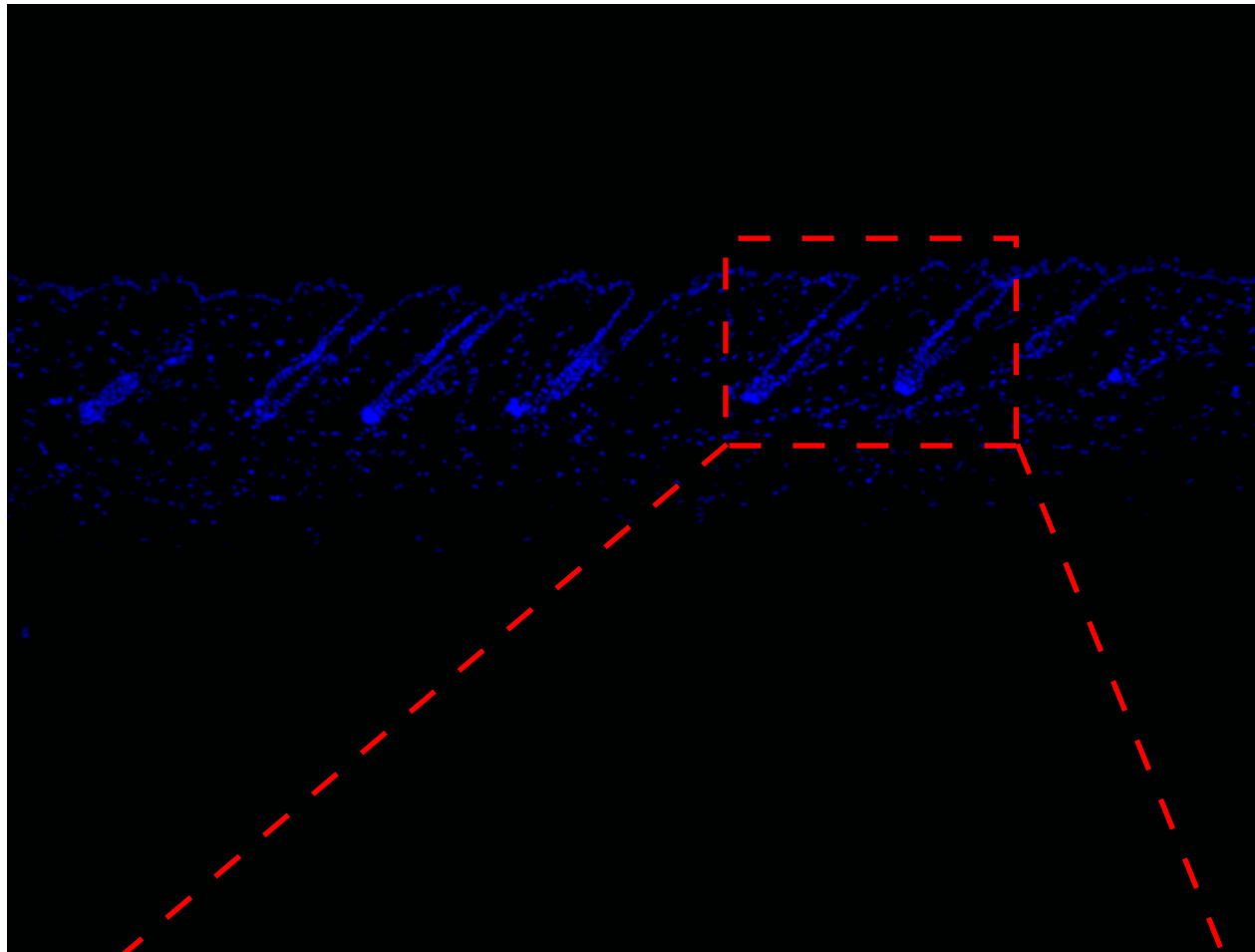

Ki67 DAPI

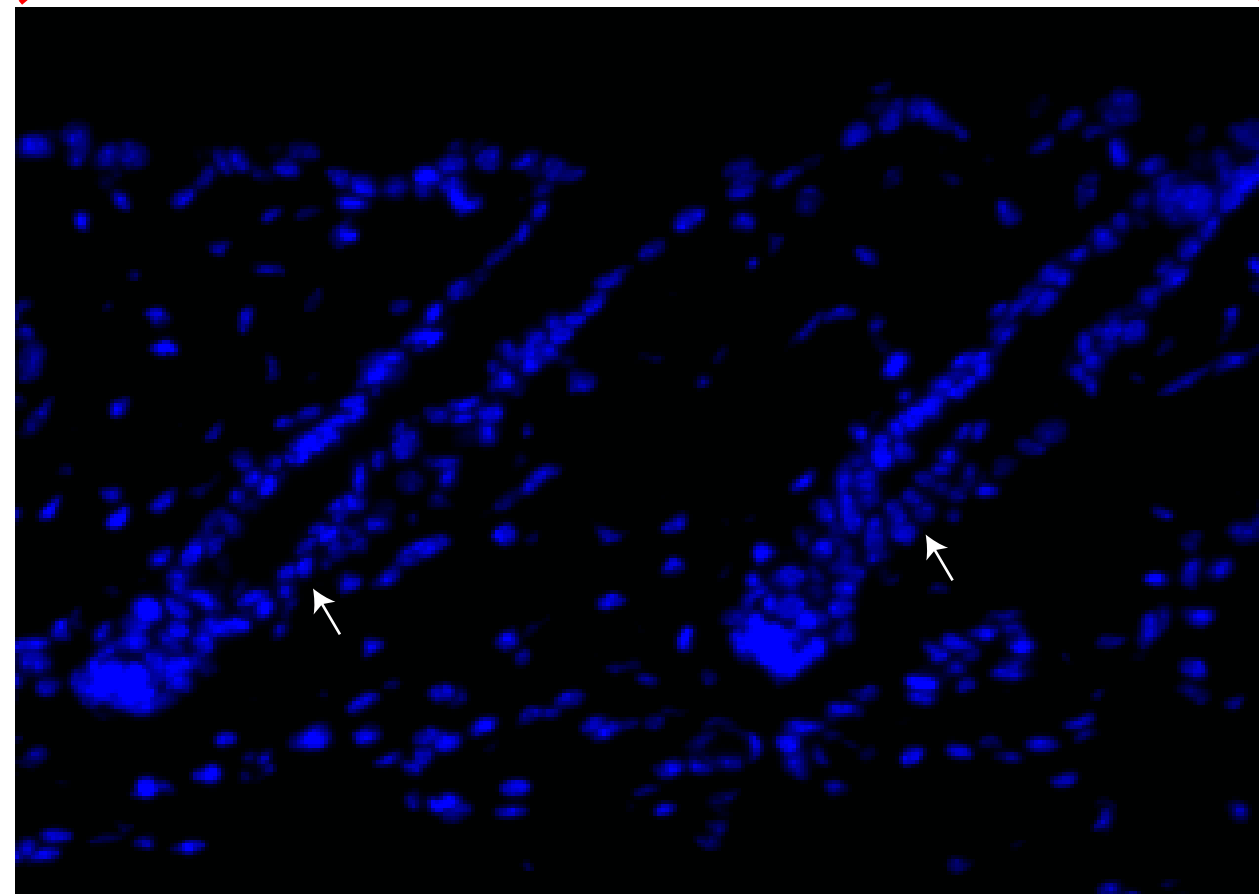

anagen

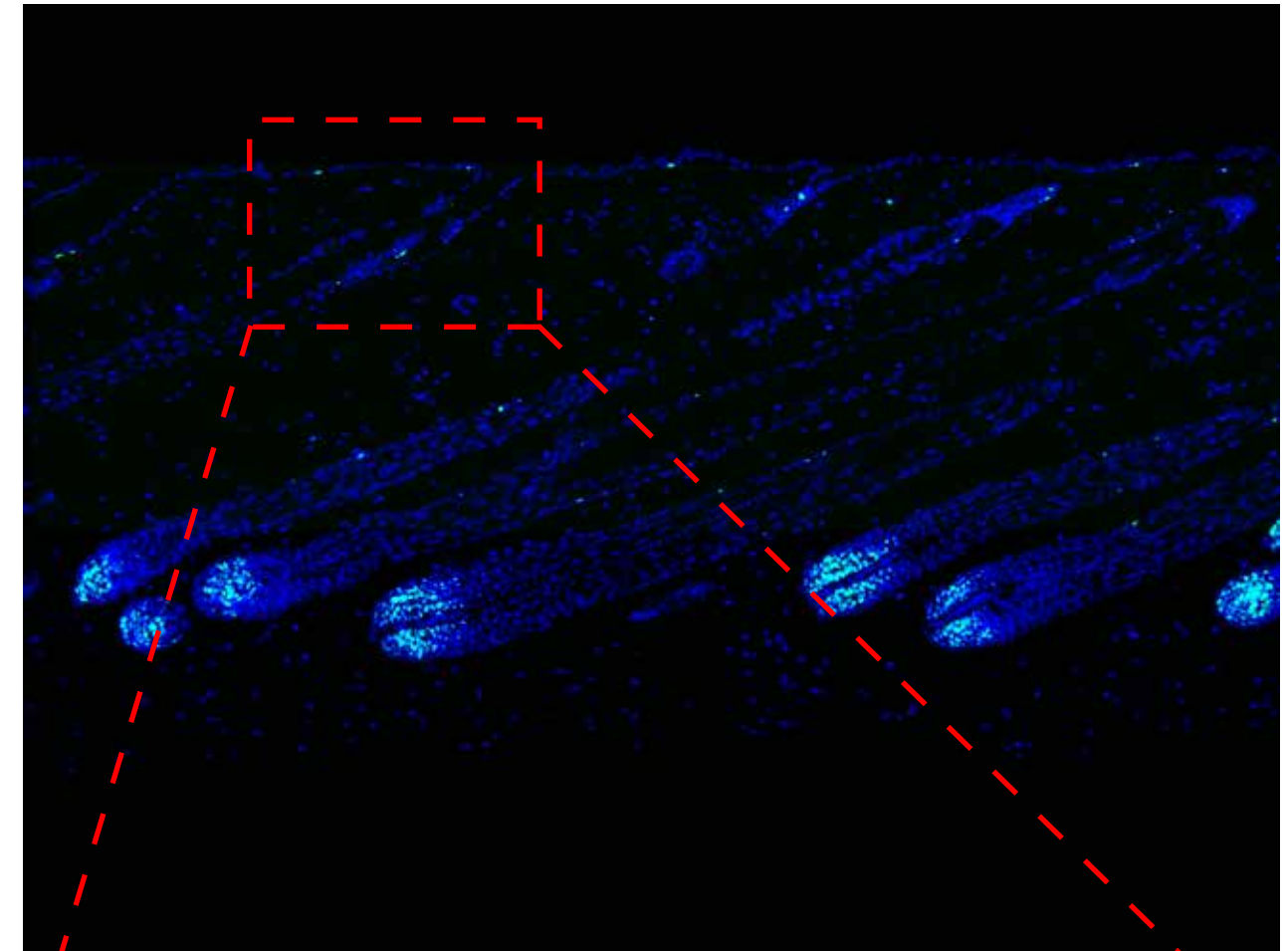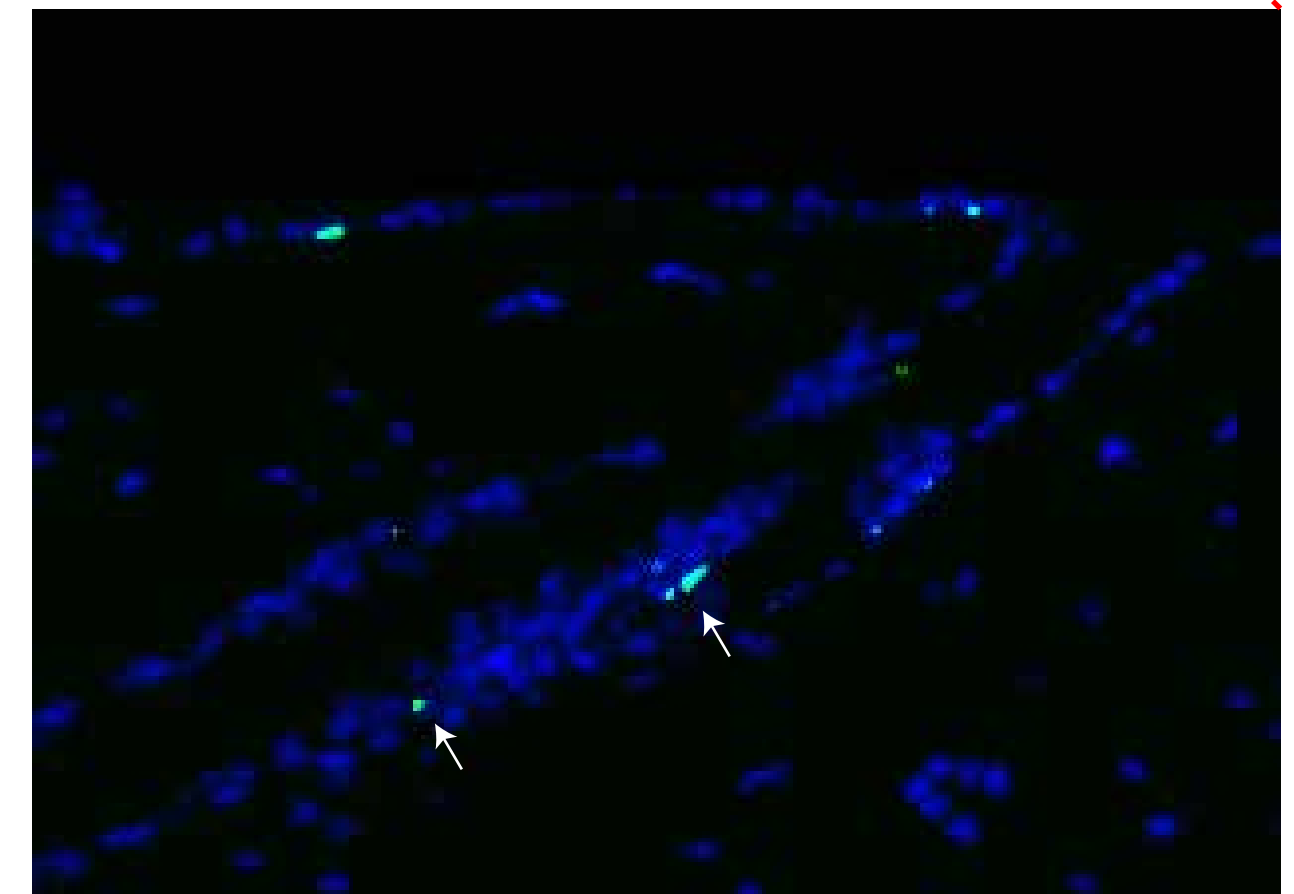

Supplement: Supplementary file 2 — Additional file 2: Fig S1. Ki67 immunofluorescence staining of hair follicle telogen and anagen. Ki67 expression was significantly upregulated in HFSC (white arrow) during anagen, indicating that the HFSC is active. [file 13578_2023_1177_MOESM2_ESM.pdf]

A

LC3B

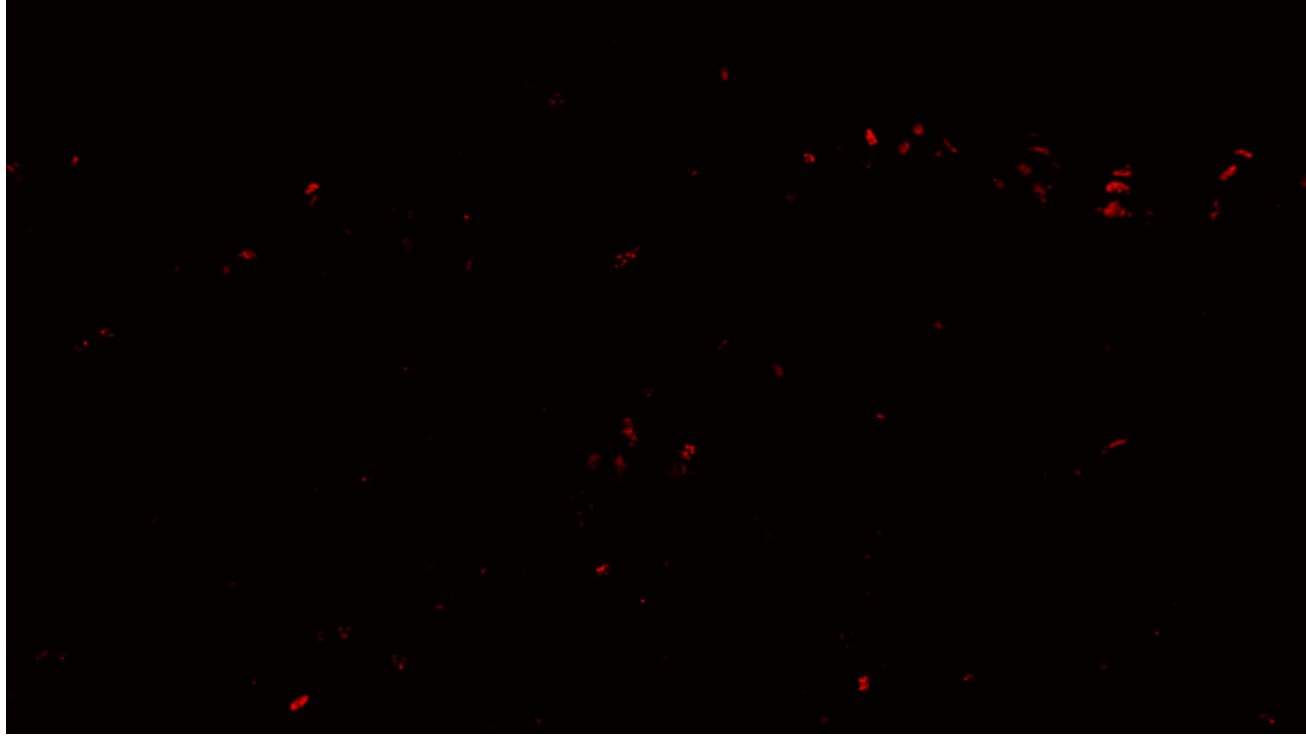

DAPI

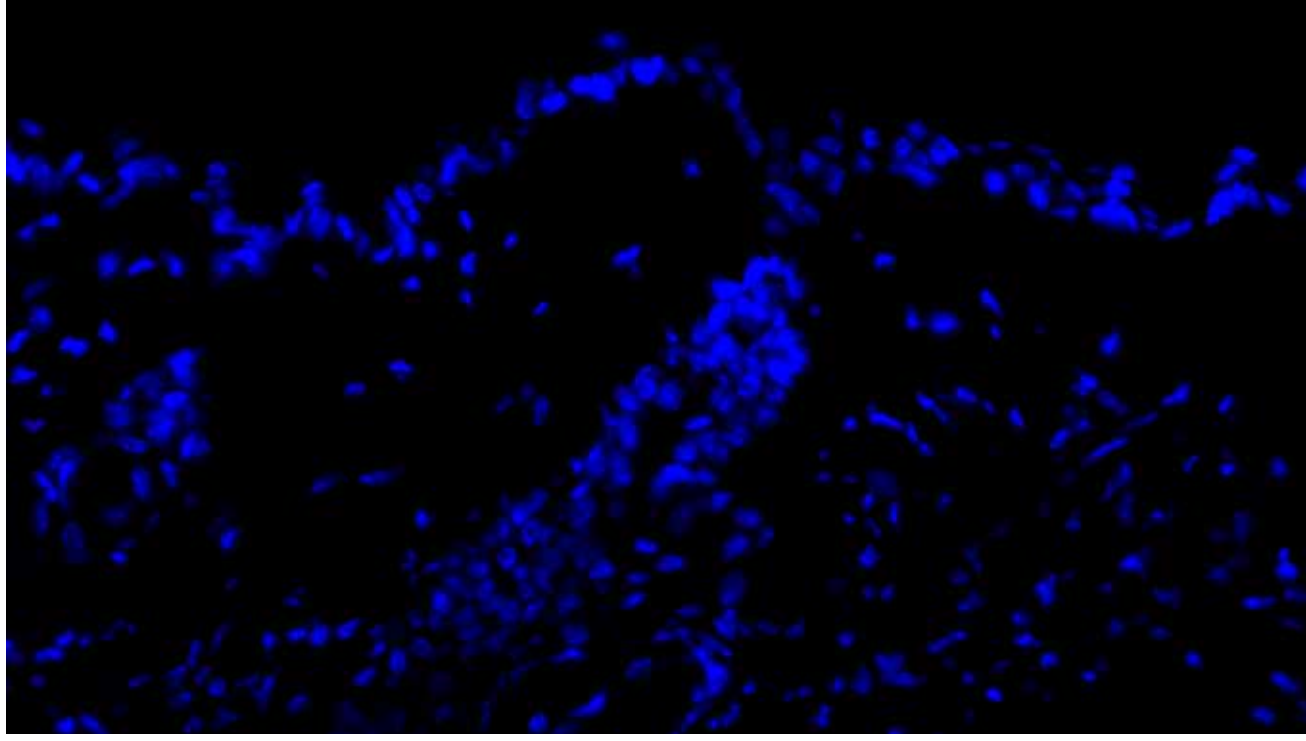

K15

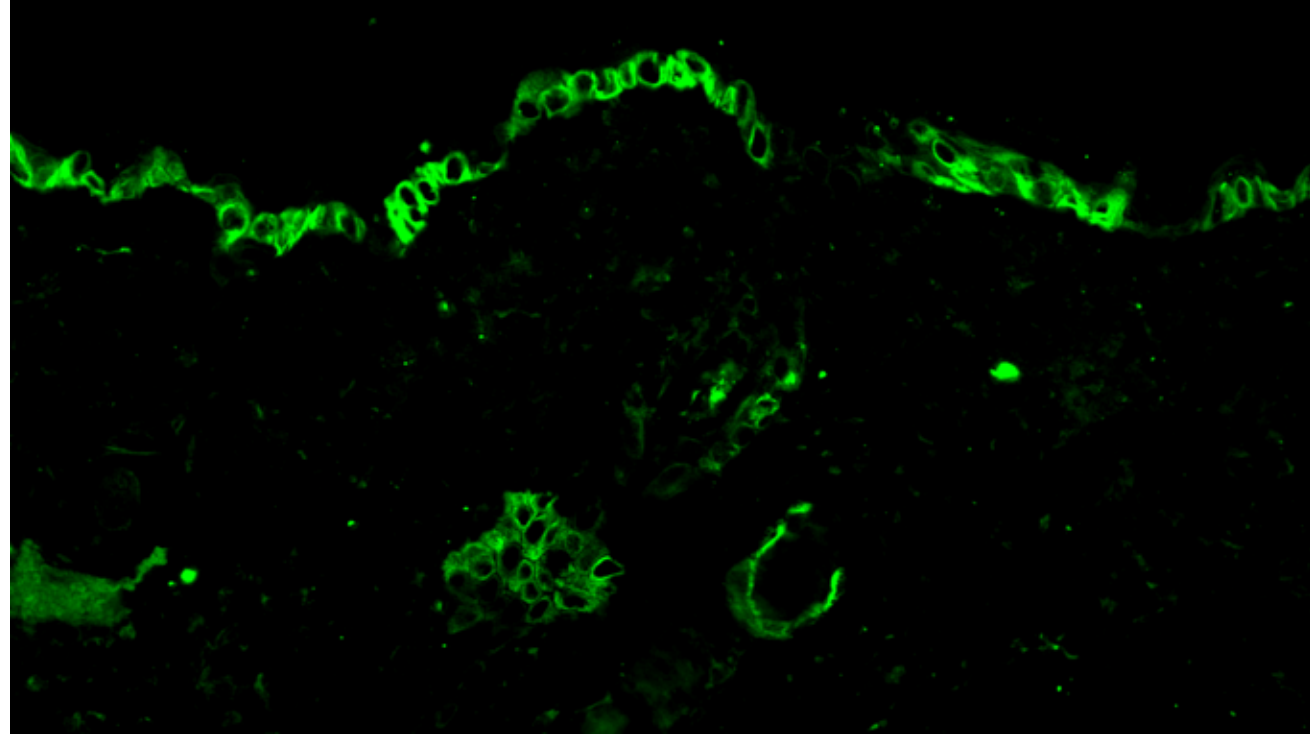

Merge

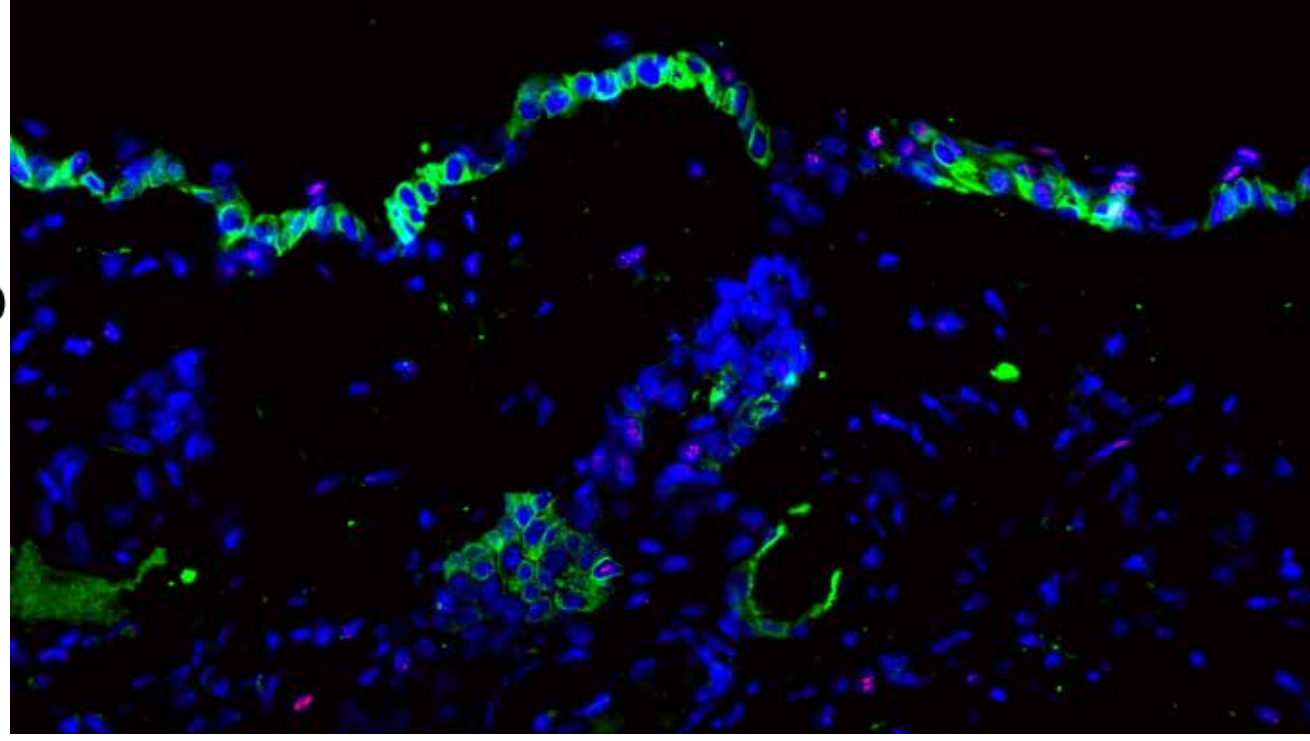

B

LC3B

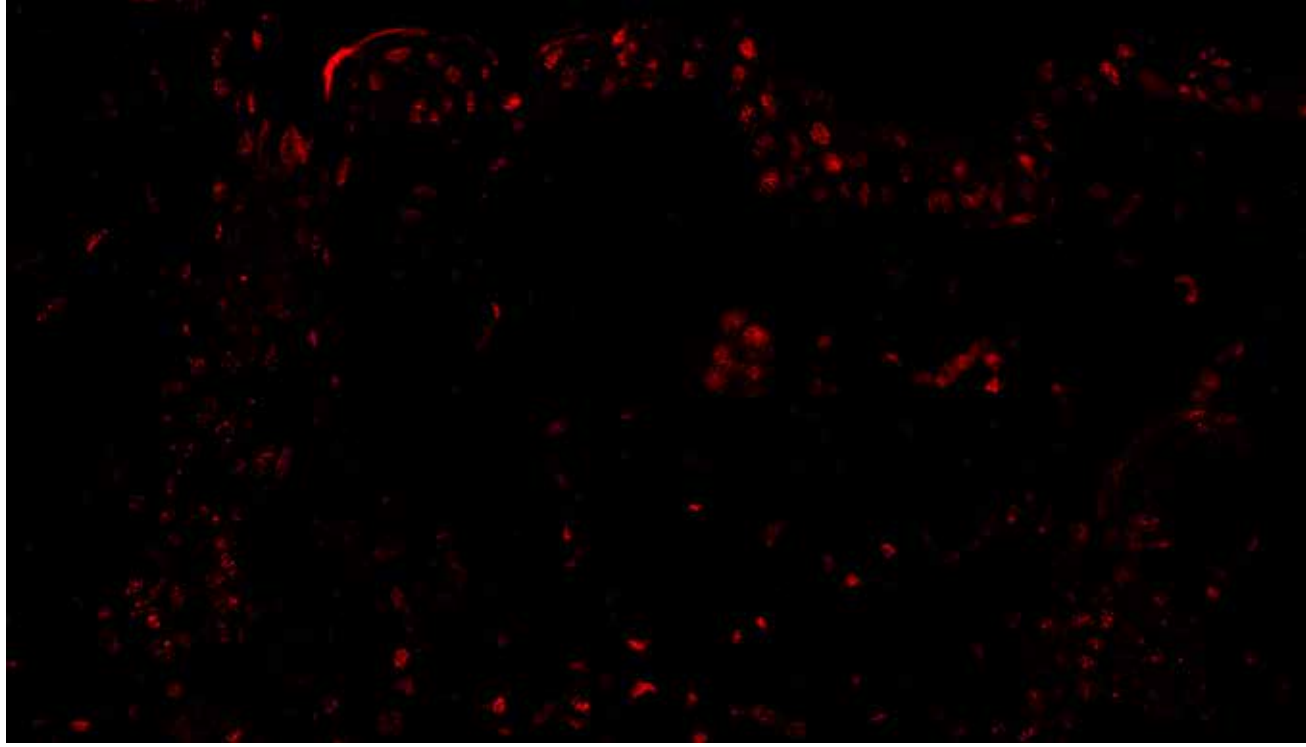

DAPI

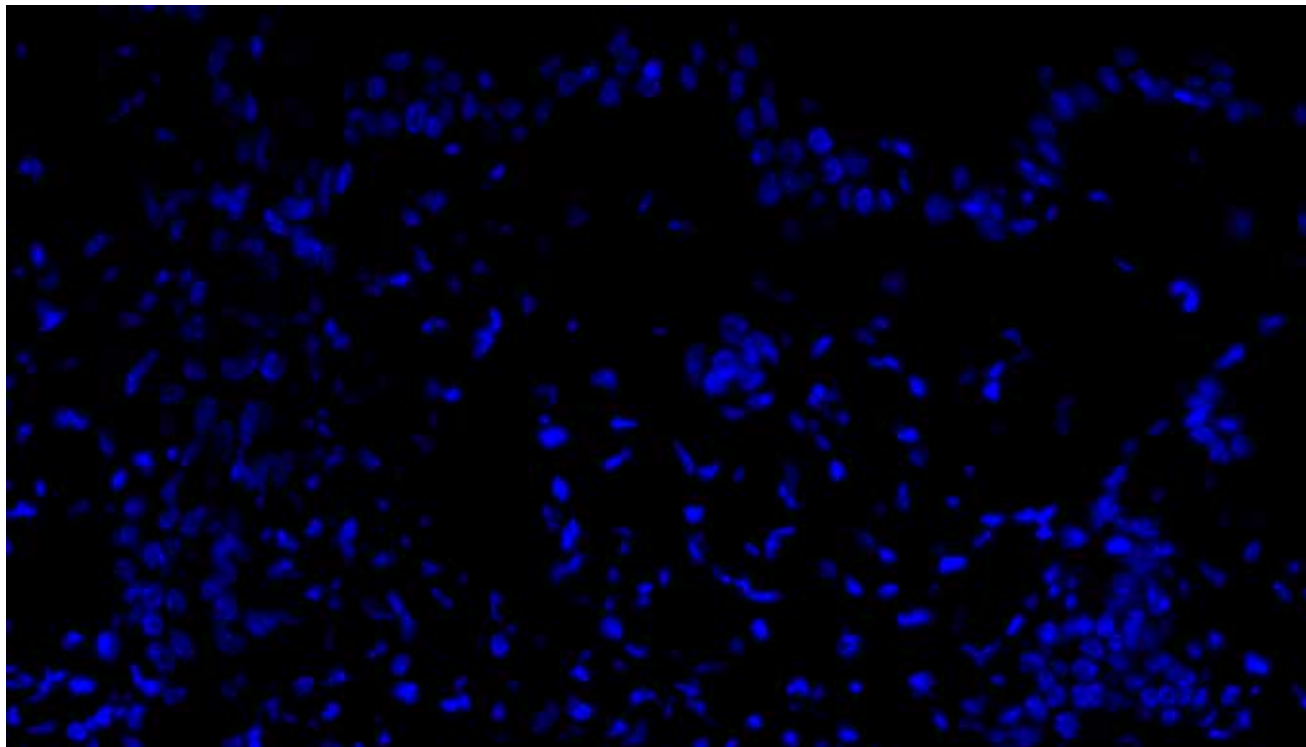

K15

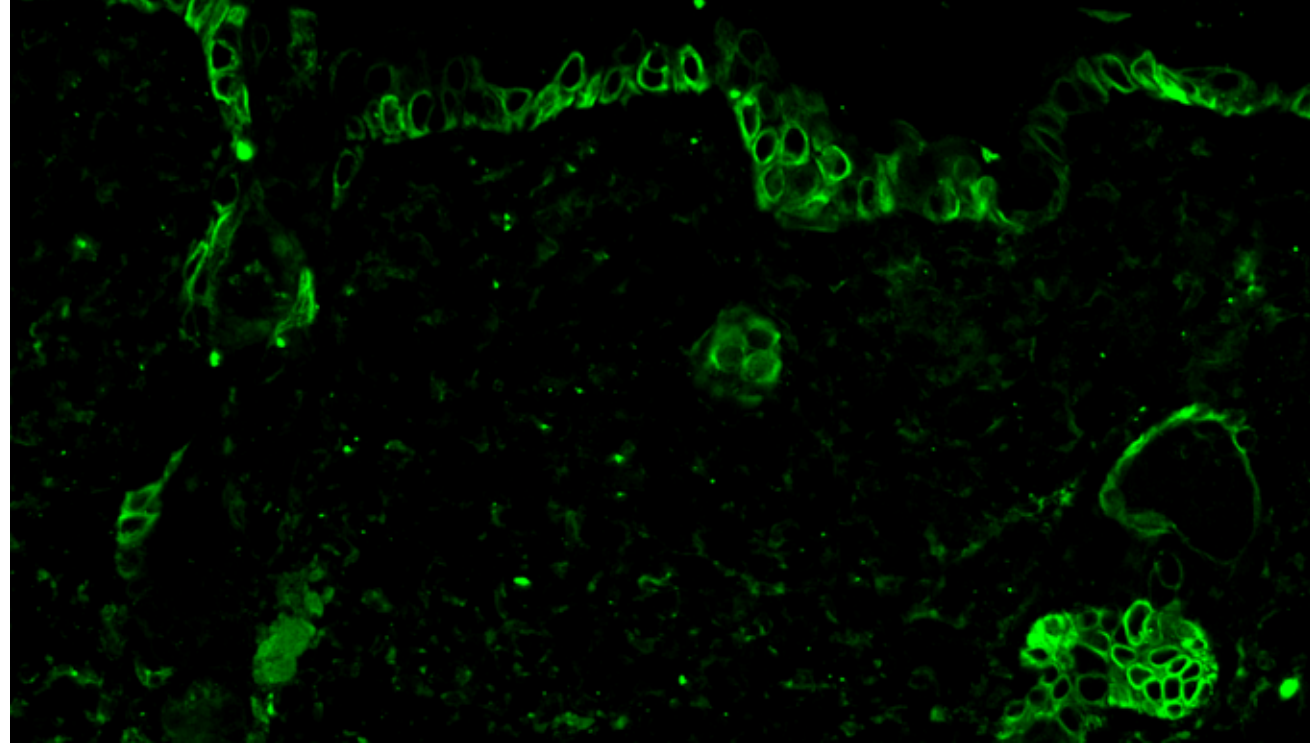

Merge

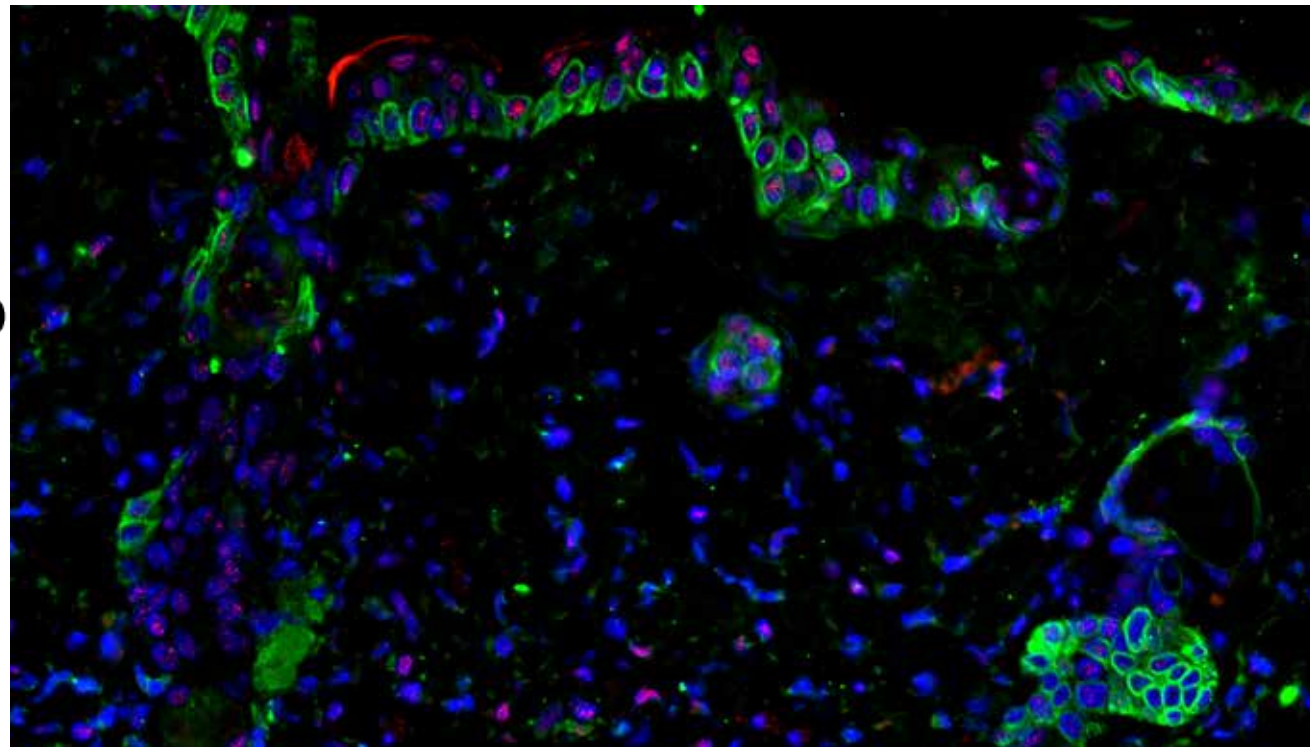

Supplement: Supplementary file 3 — Additional file 3: Fig S2. A Representative immunofluorescence images of dorsal hair follicles stained with anti-LC3B antibody(red) and anti-K15 antibody(green) after plucking hair follicles for 24 h. B Representative immunofluorescence images of dorsal hair follicles stained with anti-LC3B antibody(red) and anti-K15 antibody (green) after plucking hair follicles and topically treated with CQ for 24 h. [file 13578_2023_1177_MOESM3_ESM.pdf]

A

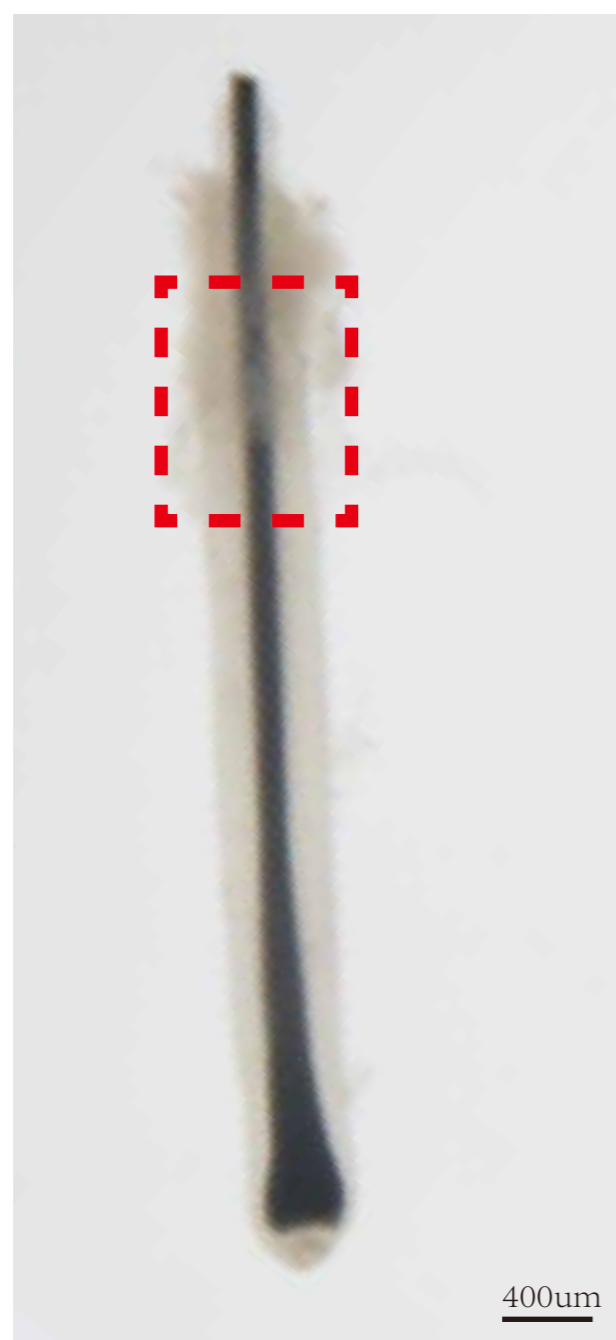

B

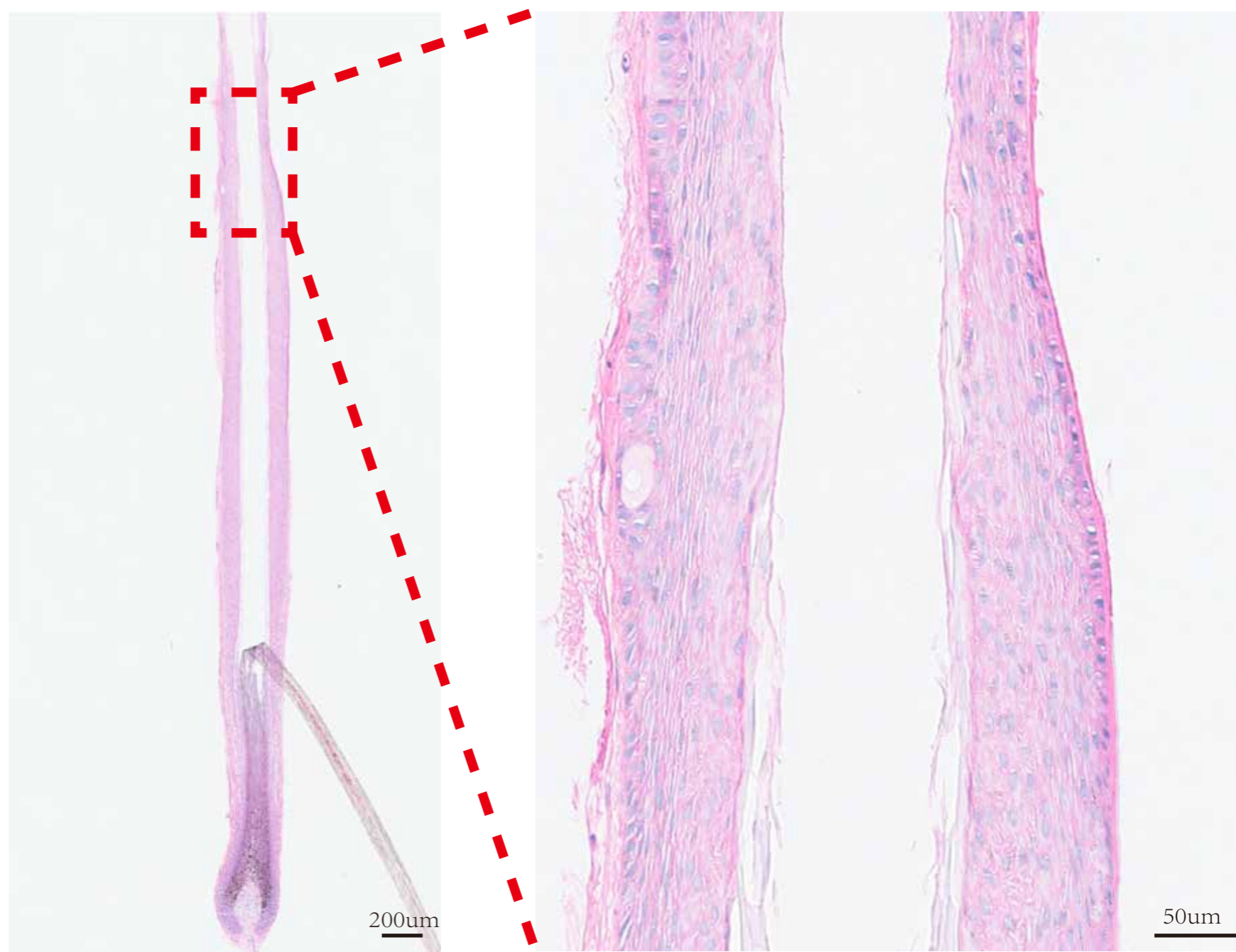

D

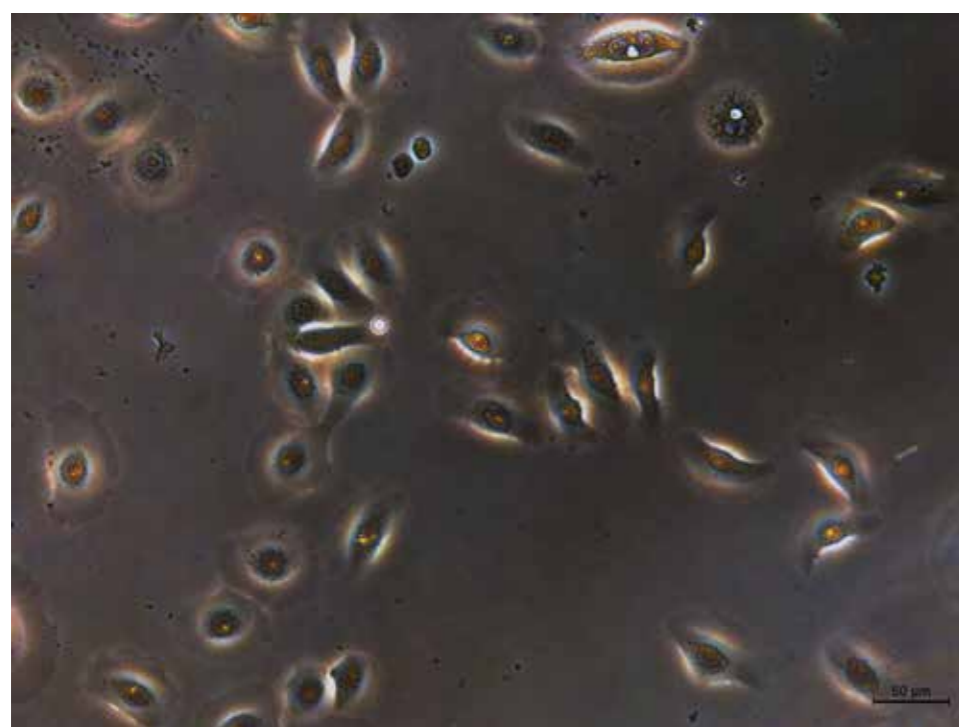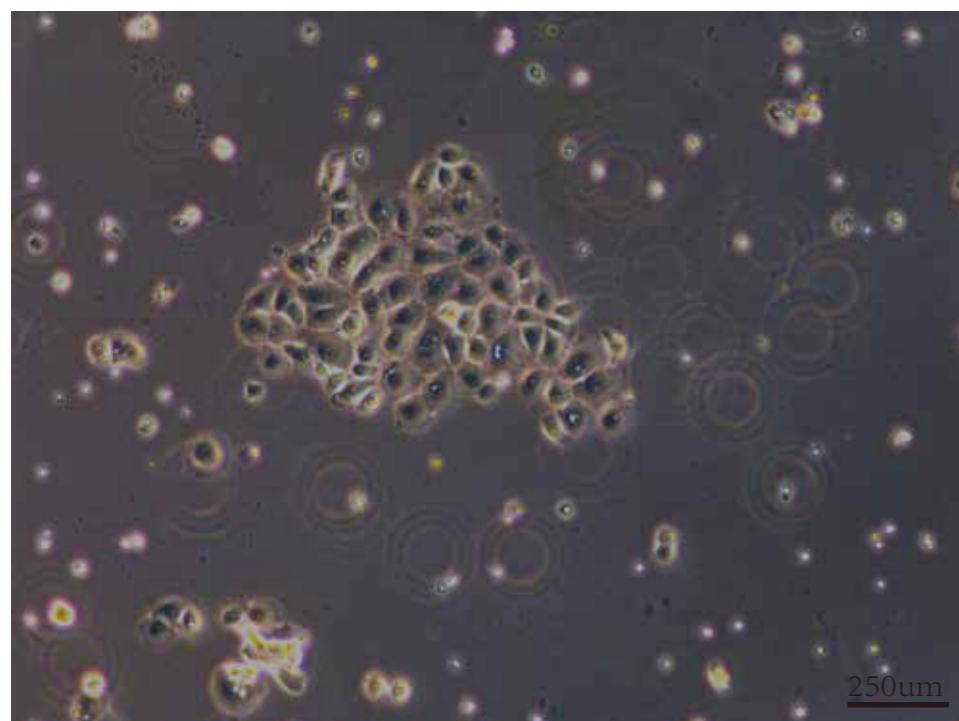

C

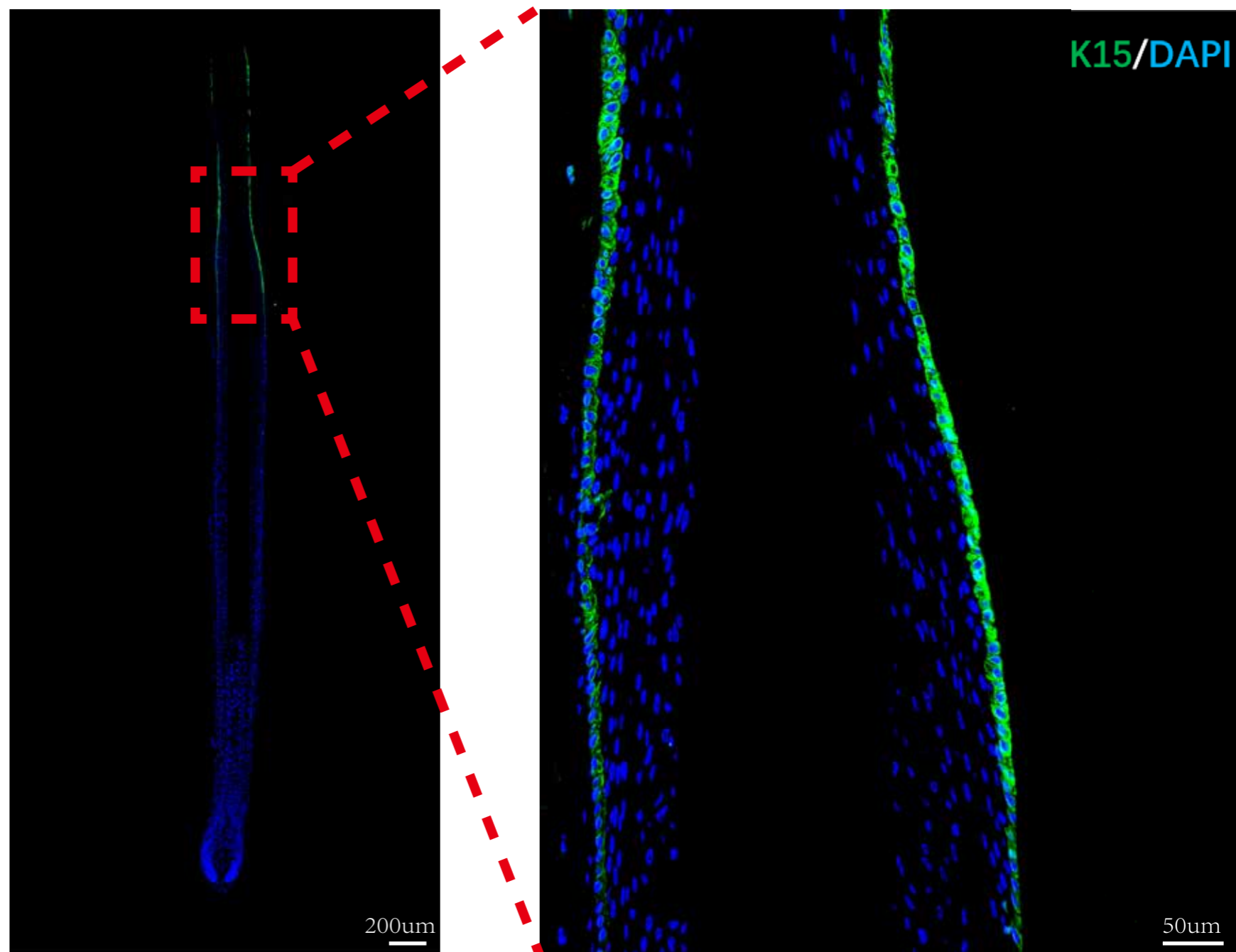

E

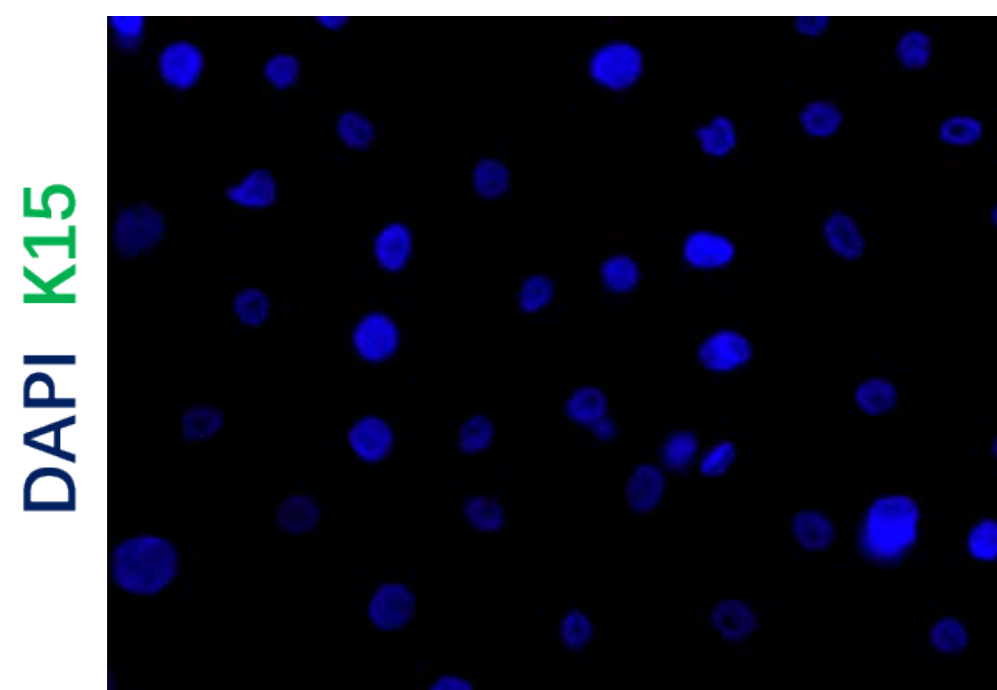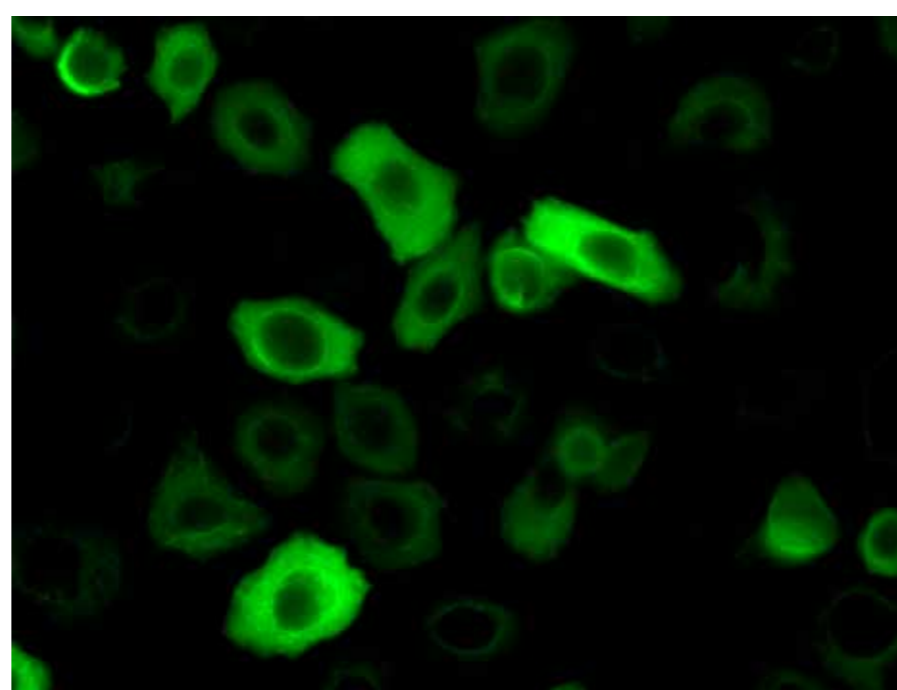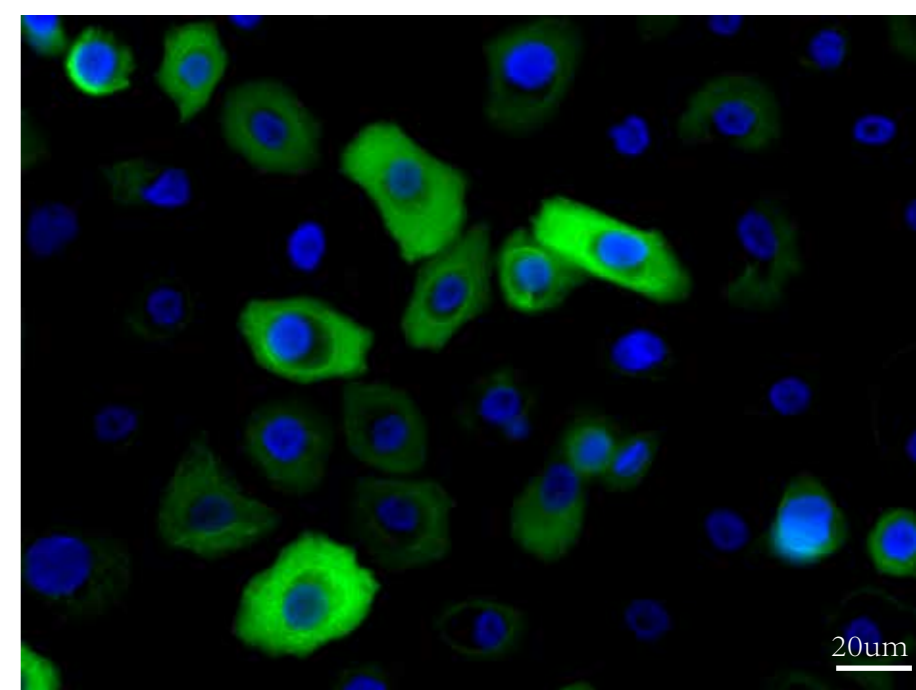

Supplement: Supplementary file 4 — Additional file 4: Fig S3. Characteristics of HFSCs obtained from the bulge of human hair follicles. A Morphology of single human hair follicle. B Microphotographs of H&E stained human hair follicle. C Immunofluorescence images of HFSCs stained with anti-K15 antibody(green) showing the location of bulge zone. D Morphology of human HFSCs cultured for 3 days(up) and 5 days(down). E Representative immunofluorescence images of human HFSCs stained with anti-K15 antibody(green). [file 13578_2023_1177_MOESM4_ESM.pdf]

A

Control

3-MA

Rapa

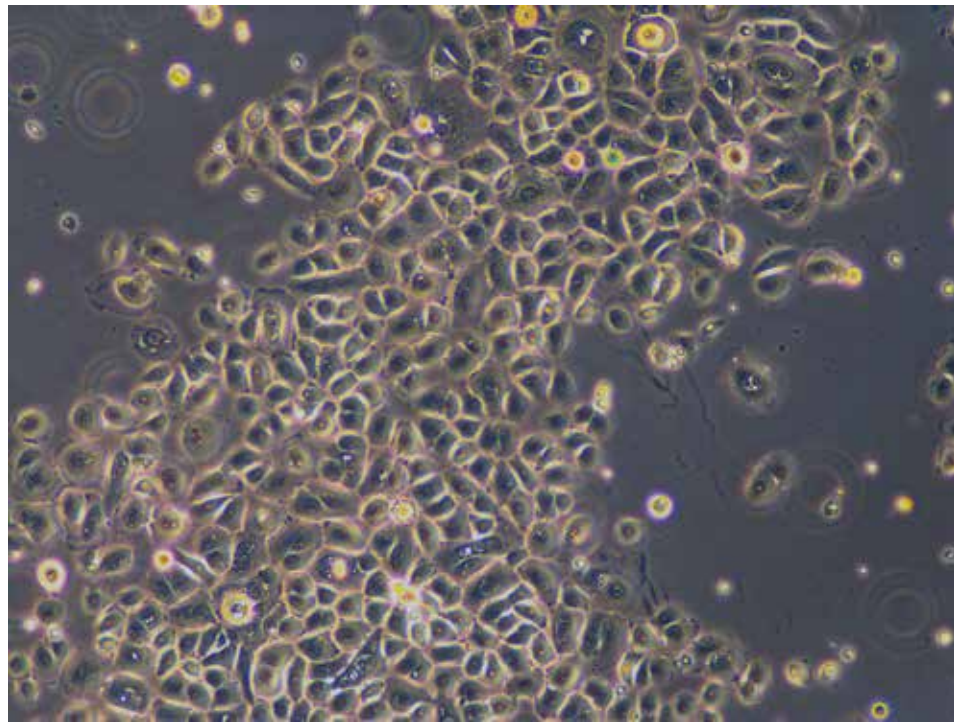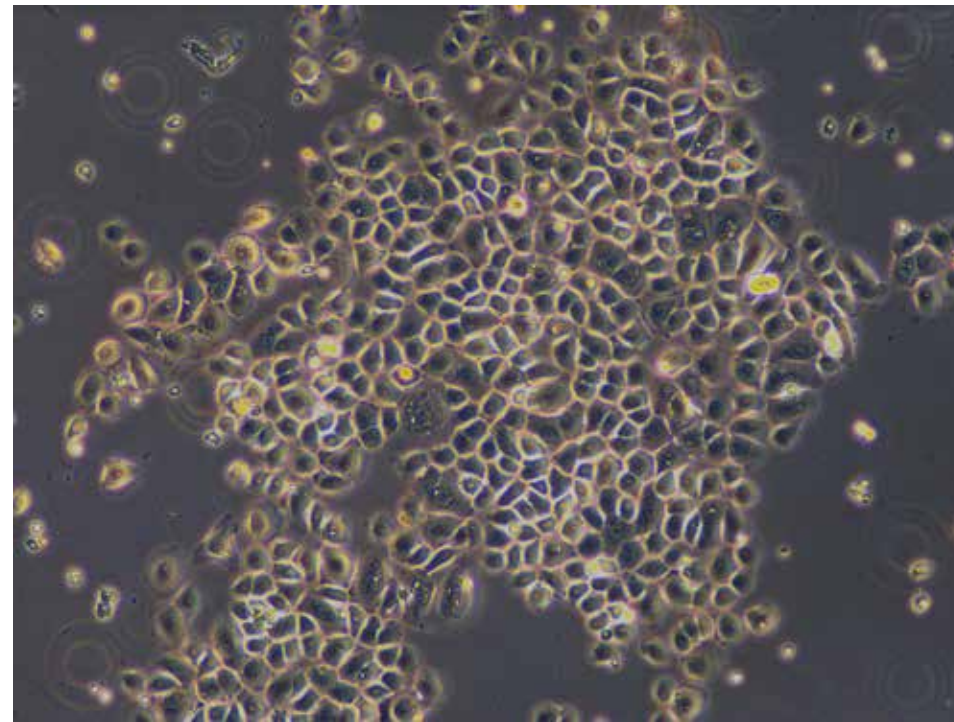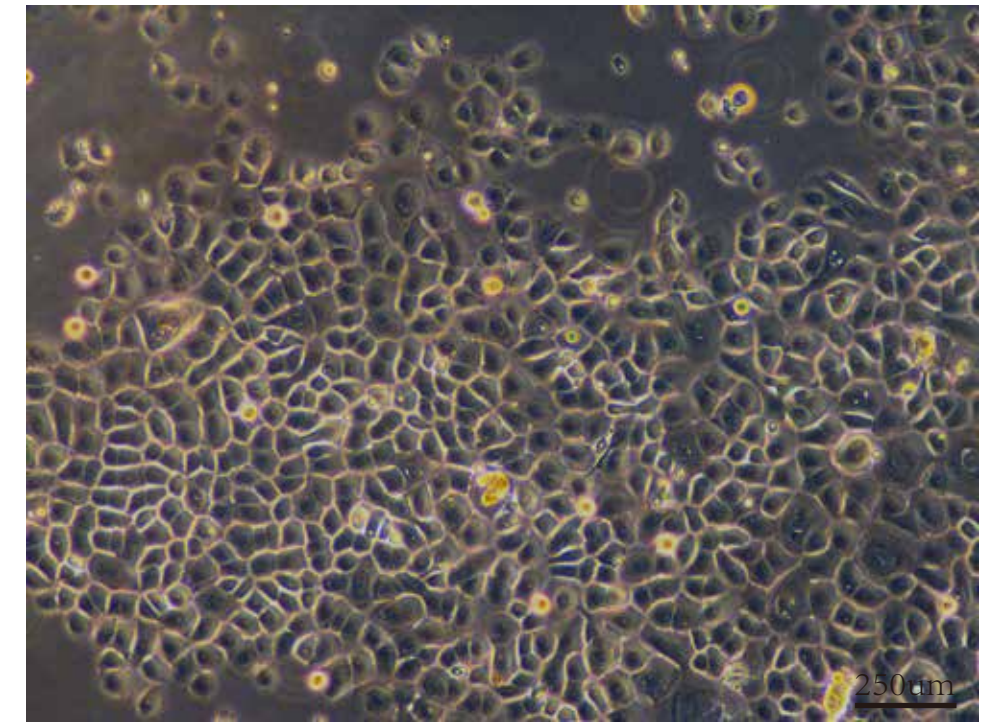

B

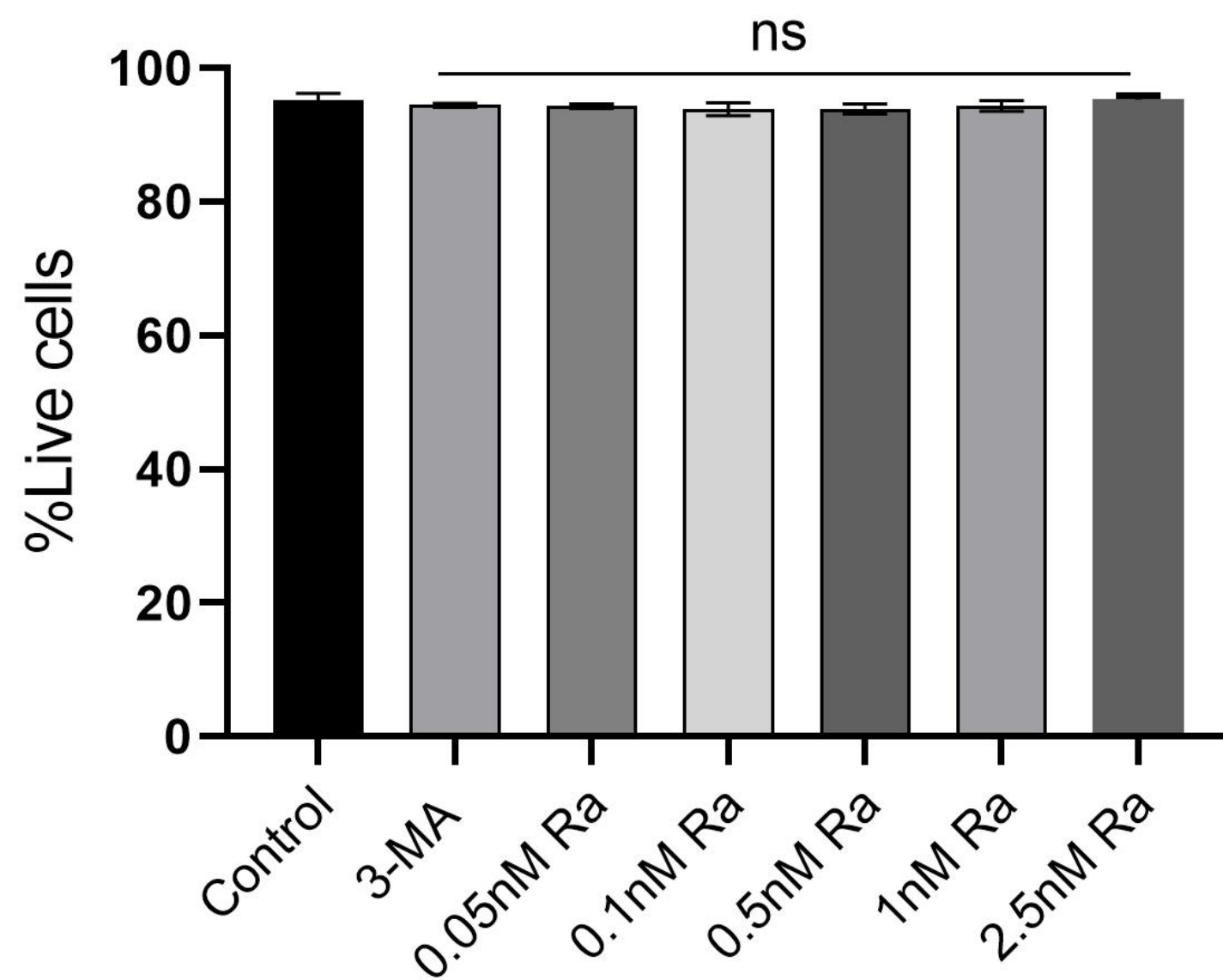

C

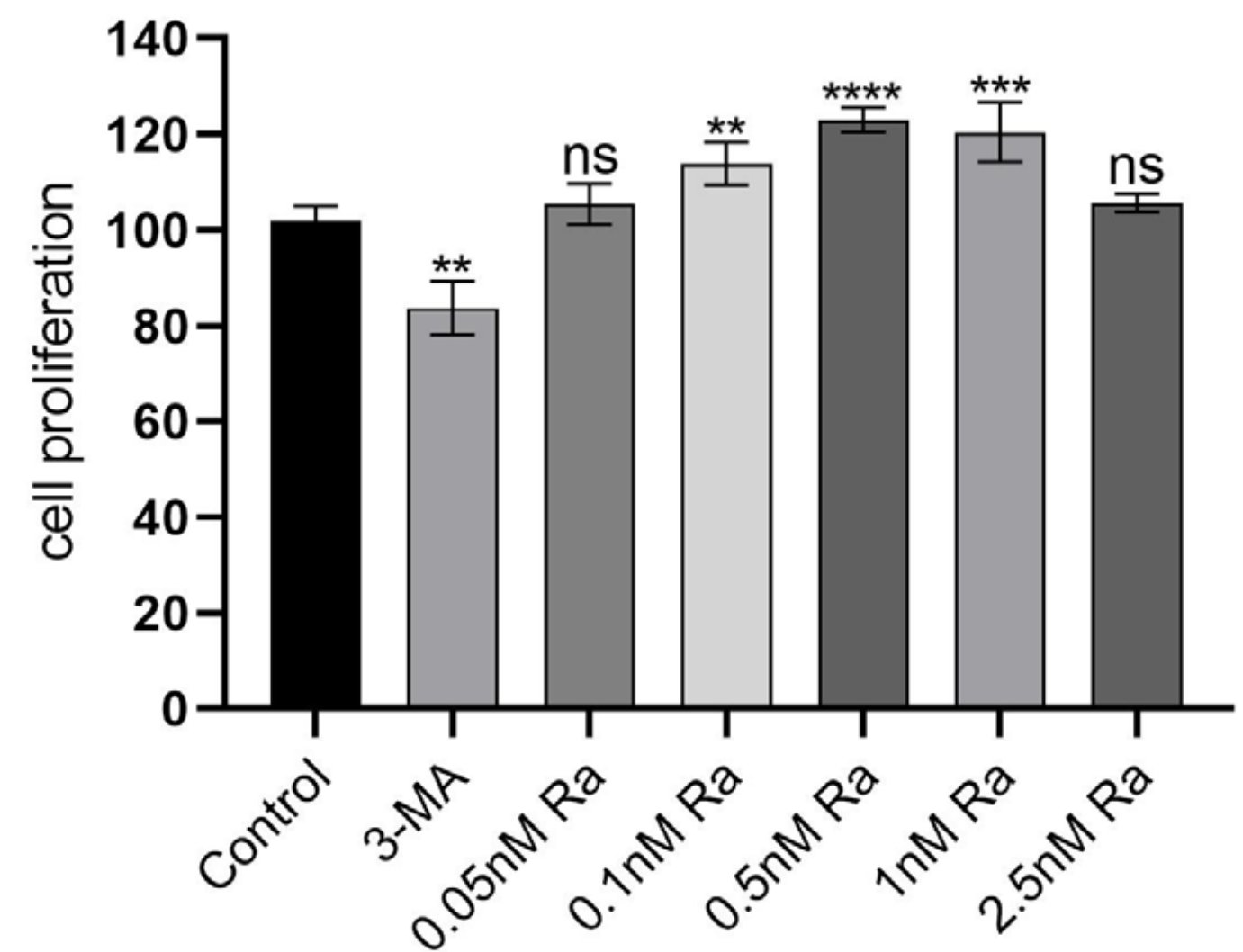

Supplement: Supplementary file 5 — Additional file 5: Fig S4. A HFSCs phenotypes after cultured with control, 3-MA or Rapa for 5 days. B Analysis of live/dead staining of HFSCs cultured with control, 3-MA or different concentrations of Rapa for 24 h. C Analysis of CCK8 assay of HFSCs cultured with control, 3-MA or different concentrations of Rapa for 24 h. The data represent the means ± S.E.M from at least three independent experiments. **P < 0.01, ***P < 0.001, ****P < 0.0001, determined by Student’s t-test, 3-MA or different concentrations of Rapa versus Control. [file 13578_2023_1177_MOESM5_ESM.pdf]
